# Supplementary material for: A primate-specific retroviral enhancer wires the XACT lncRNA into the core pluripotency network in humans
Source: Nat Commun. 2019 Dec 11;10:5652. doi: 10.1038/s41467-019-13551-1 (PMC6906429; doi:10.1038/s41467-019-13551-1)
Supplement: Supplementary file 2 — Supplementary Information [file 41467_2019_13551_MOESM2_ESM.pdf]

Supplementary figure 1

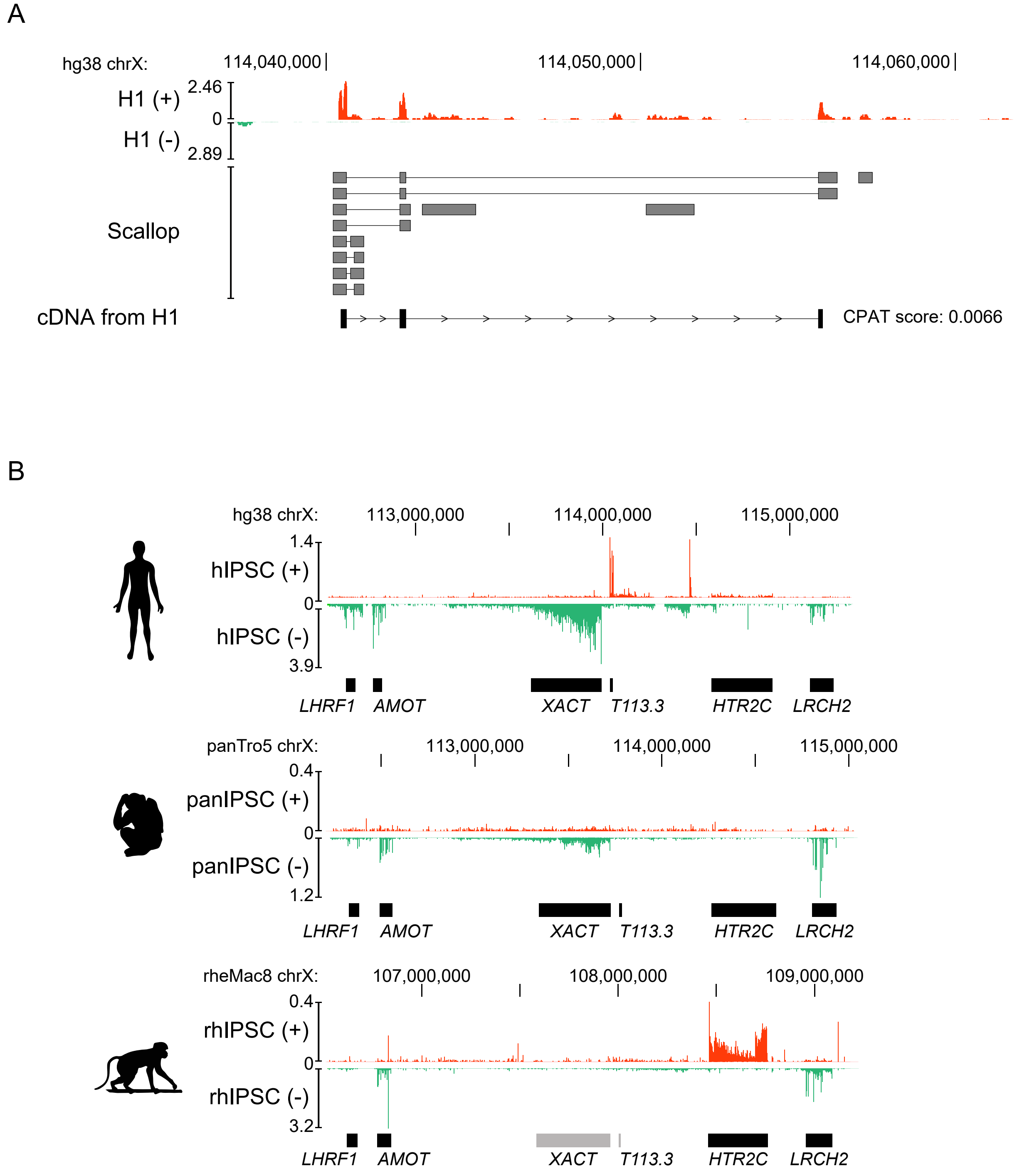

Supplementary Figure 1 – The transcriptional landscape of the *XACT*/*T113.3* locus is different across primate species

A. ENCODE strand-specific RNA-seq from male H1 hESCs (GSM758573) showing transcription over the *T113.3* gene from the plus and minus strand. Scallop transcript assembler was used to reconstruct potential transcripts from the above RNA-seq dataset and are represented in gray. cDNA cloning and sequencing from H1 hESCs is represented bellow. CPAT (Coding-Potential Assessment Tool) was used to predict the coding potential of the *T113.3* transcript and the assessed coding probability is represented.

B. International Human Epigenome Consortium (IHEC) strand-specific RNA-seq from human, chimpanzee and rhesus macaque iPSCs across the region spanning the *LHFPL1* and *LRCH2* genes[1,2]. Schematic representation of the genes found across the locus are shown below each panel. An additional uncharacterized transcript expressed from the sense strand can be observed downstream of *T113.3*, before the *HTR2C* gene. This transcript is, similarly to *T113.3*, a lncRNA derived from a HERVH retroelement that is conserved in higher primates. Syntenic regions for the human *XACT* and *T113*.3 genes are shown for rhesus macaque as grey rectangles.

Supplementary figure 2

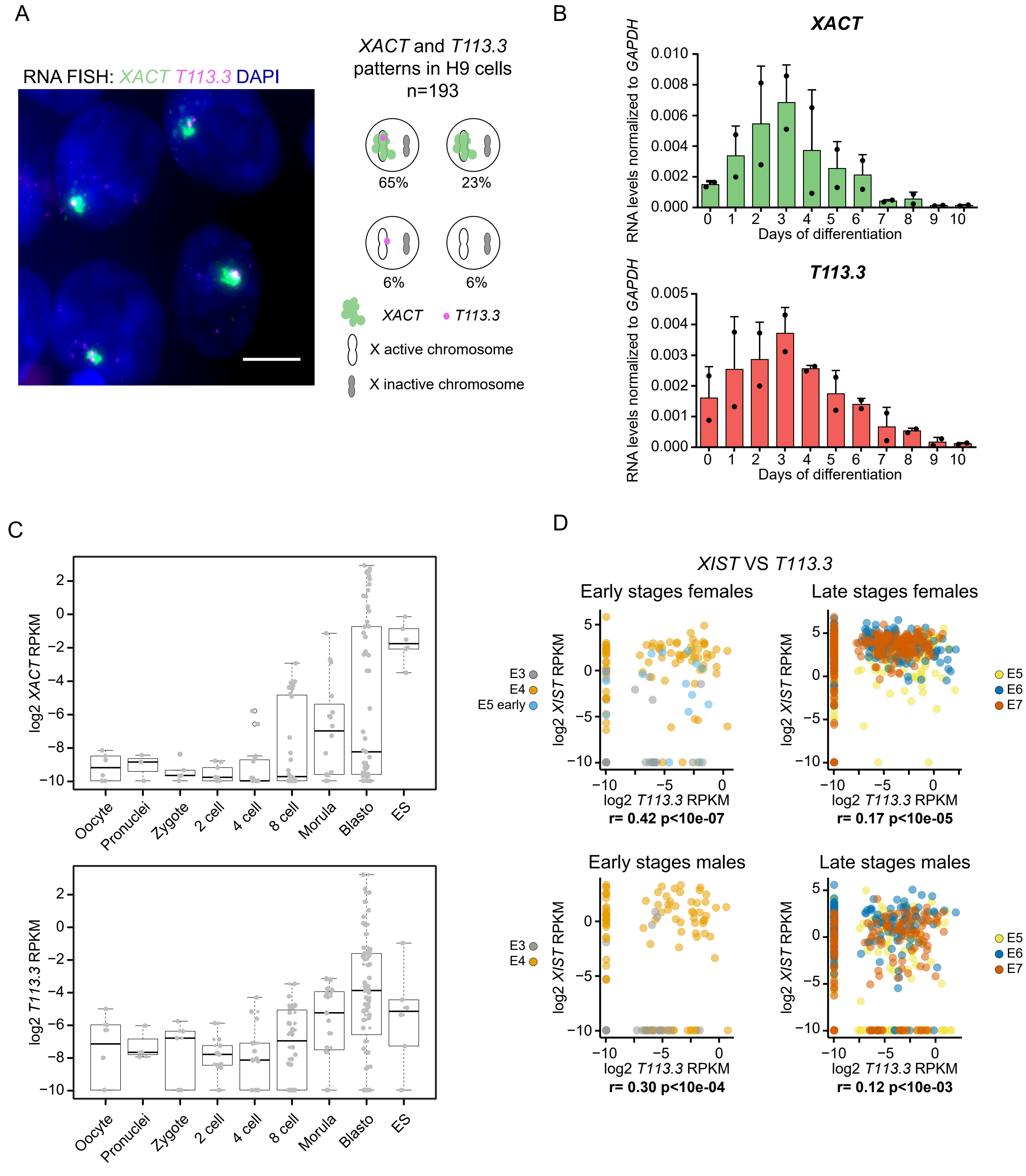

Supplementary Figure 2 – *XACT*, but not *XIST*, shows a similar expression dynamics to *T113.3* in pluripotent contexts

A. Analysis of *XACT* (green) and *T113.3* (magenta) expression by RNA-FISH in female H9 hESCs. Percentage of cells co-expressing *XACT* and *T113.3* from the same locus is indicated. Quantification of the different patterns of expression is represented on the right (n=193). The white scale bar represents 5 µm.

B. Quantitative RT-PCR analysis of *XACT* and *T113.3* expression during a 10-day undirected differentiation of female WIBR2 hESCs. The bar charts correspond to the average of two independent differentiation experiments. Error bars indicate the s.d. The correlation coefficient for the expression dynamics of the two genes is shown (r= 0.97, Pearson correlation).

C. Boxplot of *XACT* and *T113.3* expression levels (log2 RPKM) according to developmental stage. Dataset from[3–5].

D. Plot of *T113.3* versus *XIST* expression levels (log2 reads per kilobase per million mapped reads [RPKM]) in early (E3, E4 and early E5) and late stage (E5, E6 and E7) female (upper panels) and male cells (lower panels), with corresponding Spearman correlation score and p-value. Dataset from[6].

Supplementary figure 3

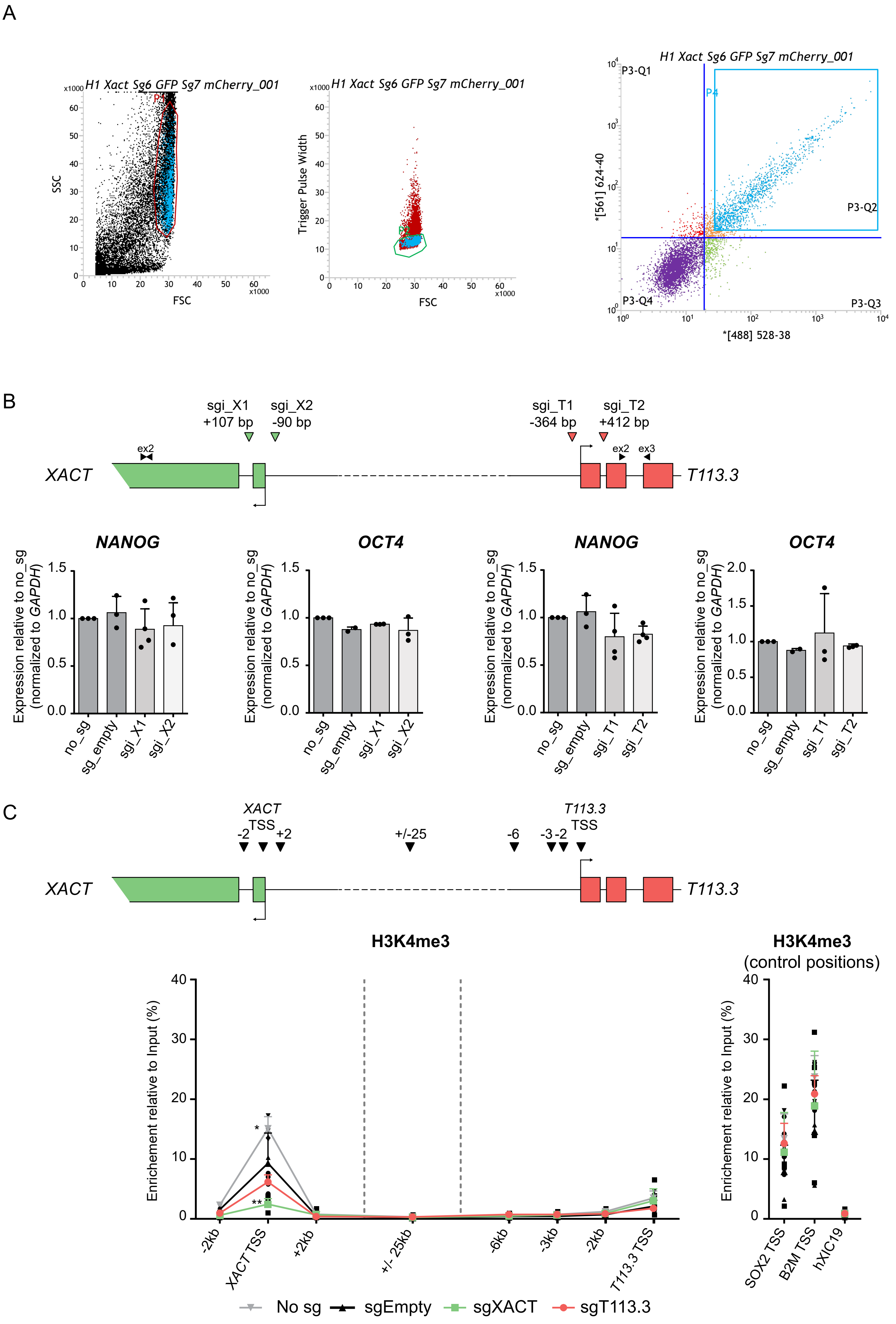

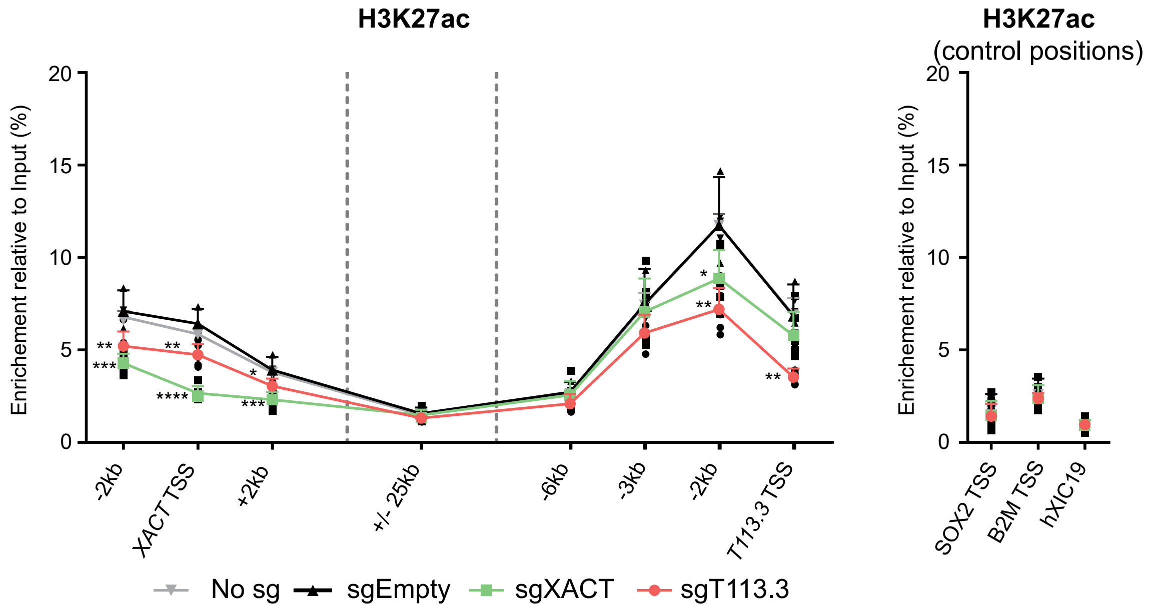
H3K27ac
H3K27ac
(control positions)
Enrichement relative to Input (%)
Enrichement relative to Input (%)
20
15
10
5
0
-2kb
XACT TSS
+2kb
+/- 25kb
-6kb
-3kb
-2kb
T113.3 TSS
SOX2 TSS
B2M TSS
hXIC19
**
***
**
****
*
***
*
**
**
No sg
sgEmpty
sgXACT
sgT113.3

Supplementary Figure 3 – Interfering with the transcription of *XACT* or *T113.3* does not impact on expression of pluripotency markers

A. General gating strategies used for cell sorting of hESCs in Figures 3, 4B and 5B. Side scatter (SSC) and forward scatter (FSC) density plots are represented on the left. A gate was applied to identify the bulk of the population of hESCs for downstream analysis. To detect and eliminate doublets, the trigger pulse width was analysed (middle panel). A new gate was defined to select for individualized cells. Fluorophore analysis plots using filters 528/38 (to detect GFP) and 624-40 (to detect mCherry) were used to detect GFP and mCherry double positive cells (right panel). A strict quadrant, to reduce the chance of false positives, was defined as the gate for cell sorting.

B. Quantification of *NANOG* and *OCT4* steady state RNA levels by quantitative RT-PCR upon *XACT* CRISPRi (left bar charts) and *T113.3* CRISPRi (right bar charts) in H1 hESCs. Error bars represent the s.d. of at least 3 different passages of hESCs infected with guides targeting either *XACT* or *T113.3*.

C. ChIP-qPCR analysis of H3K4me3 (top graphs) and H3K27ac (bottom graphs) enrichment across the *XACT*/*T113.3* locus upon *XACT* CRISPRi (green squares) or *T113.3* CRIPRi (red circles). The positions analysed are indicated with arrows. ChIP-qPCR data for untransfected cells (gray inverted triangles) and cells transfected with a vector without a sgRNA (black triangles) are also shown. Right graph represents the relative enrichment of each mark in control positions (SOX2 TSS, B2M TSS and hXIC19). Error bars represent the s.d. of 3 passages of each of 2 independent hESC lines infected with different guides targeting either *XACT* or *T113.3* (n=6). Statistical significances were determined using a 1-way ANOVA test (all conditions compared to sg_empty). P-values: < 0,05 (*), <0,01 (**), <0,001 (***) and <0,0001 (****).

Supplementary figure 4

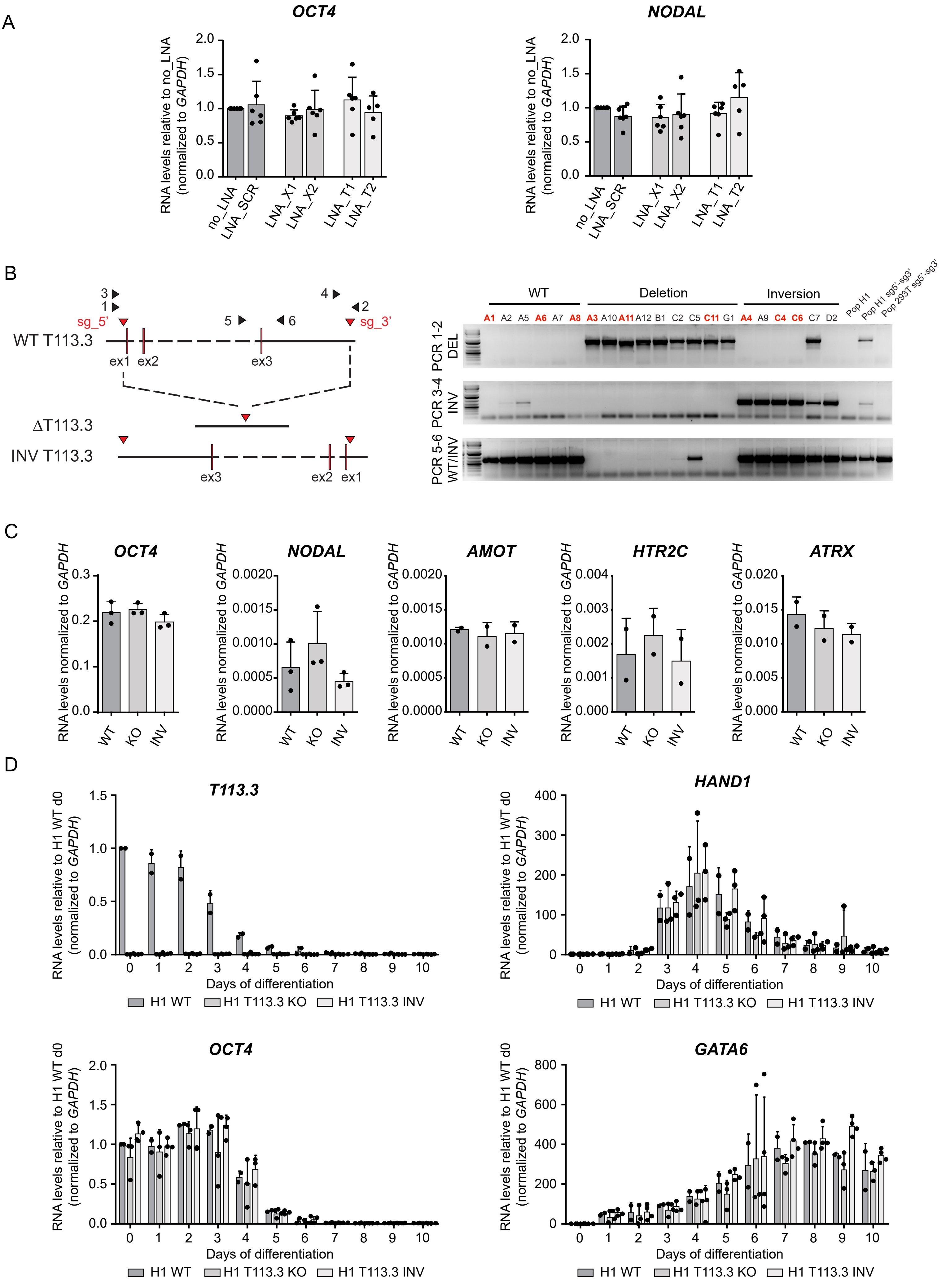

Supplementary Figure 4 – The LTR7/HERVH *T113.3* is dispensable for the transcriptional regulation of the *XACT*/*T113.3* locus in pluripotent cells and during undirected differentiation of H1 hESCs

A. Quantification of the global RNA levels of *OCT4* (left bar chart) and *NODAL* (right bar chart) by RT-qPCR upon *XACT* LNA gapmers or *T113.3* LNA gapmers KDs. The bar charts correspond to the average of at least 5 replicates per condition.

B. Schematic representation of the *T113.3* deletion strategy (left panel), showing the positions of the CRISPR sgRNAs (red triangles) and screening primers used to characterize the clones (black arrows). The right panels represent electrophoretic gel pictures showing the PCR amplicons obtained with the different screening primers on genomic DNA from the different clones. Top panel: PCR assay detecting the deleted allele (PCR 1-2); middle panel: PCR assay detecting the inversion of the *T113.3* gene (PCR 3-4); bottom panel: PCR assay testing the presence of the *T113.3* gene (found in WT or INV clones). Selected clones are highlighted in red.

C. Quantification of the steady state RNA levels of *OCT4* (pluripotency marker), *NODAL* (differentiation marker), *AMOT*, *HTR2C* (genes flanking the *XACT*/*T113.3* locus) and *ATRX* (gene on the X chromosome) by quantitative RT-PCR in H1 *T113.3* WT, KO and INV clones. The bar charts correspond to the average of two or three independent clones.

D. Quantitative RT-PCR analysis of *T113.3*, *OCT4*, *HAND1* and *GATA6* expression during a 10-day undirected differentiation of H1 T113.3 WT, KO and INV hESC lines. n≥2.

Error bars indicate the s.d.

Supplementary figure 5

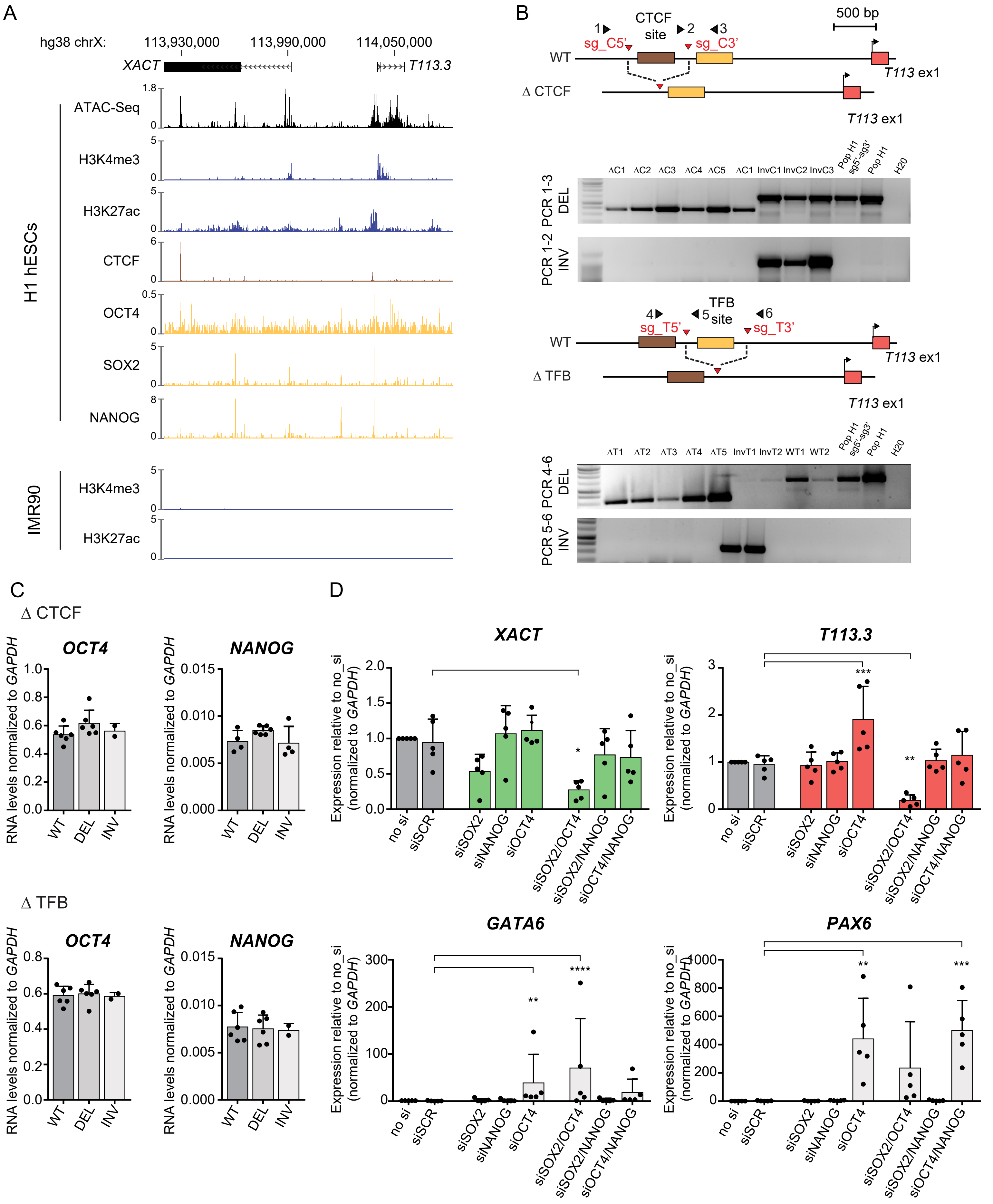

Supplementary Figure 5 – The LTR/ERV element upstream of *T113.3* is a pluripotent specific enhancer

A. ATAC-seq (black) and ChIP-seq data for H3K4me3, H3K27ac (blue), CTCF (brown), OCT4, SOX2 and NANOG (orange) enrichment across the *XACT*/*T113.3* locus in H1 hESCs (top panel). ChIP-seq data for H3K4me3 and H3K27ac enrichment in IMR90 is shown below. Publicly available data was obtained from the ENCODE project[7] and CISTROME DB[8].

B. Schematic representation of the *T113.3* locus, showing the positions of the CRISPR sgRNAs (red triangles) used for targeting of the CTCF (top scheme) or TFB sites (bottom scheme) and the primers used for genotyping (black arrows). Panels bellow each scheme represent electrophoretic gels showing the PCR amplicons obtained with the different screening primers on genomic DNA from H1 CTCF- (top) and TFB- (bottom) targeted clones. Top panels: PCR assay detecting the deleted and WT alleles (PCR 1-3 for CTCF site and PCR 4-6 for TFB site); bottom panel: PCR assay detecting inversion of the targeted region (PCR 1-2 for CTCF site and PCR 5-6 for TFB site).

C. Quantification of *OCT4* (left bar chart) and *NANOG* (right bar chart) steady state RNA levels by RT-qPCR in CTCF- (top bar charts) and TFB- (bottom bar charts) targeted H1 clones. The bar charts correspond to the average of at least, two independent clones.

D. RT-qPCR analysis of the RNA levels of *XACT* (top left bar chart), *T113.3* (top right bar chart), *GATA6* (bottom left bar chart) and *PAX6* (bottom right bar chart) in cells transfected with siRNA targetting OCT4, SOX2, NANOG or different combinations of these siRNAs. Bar charts correspond to the average of five independent experiments. Statistical significances were determined using a 1-way ANOVA test (all tests compared to siSCR). P-values: < 0,05 (*), <0,01 (**), <0,001 (***) and <0,0001 (****).

Error bars indicate the s.d.

Supplementary figure 6

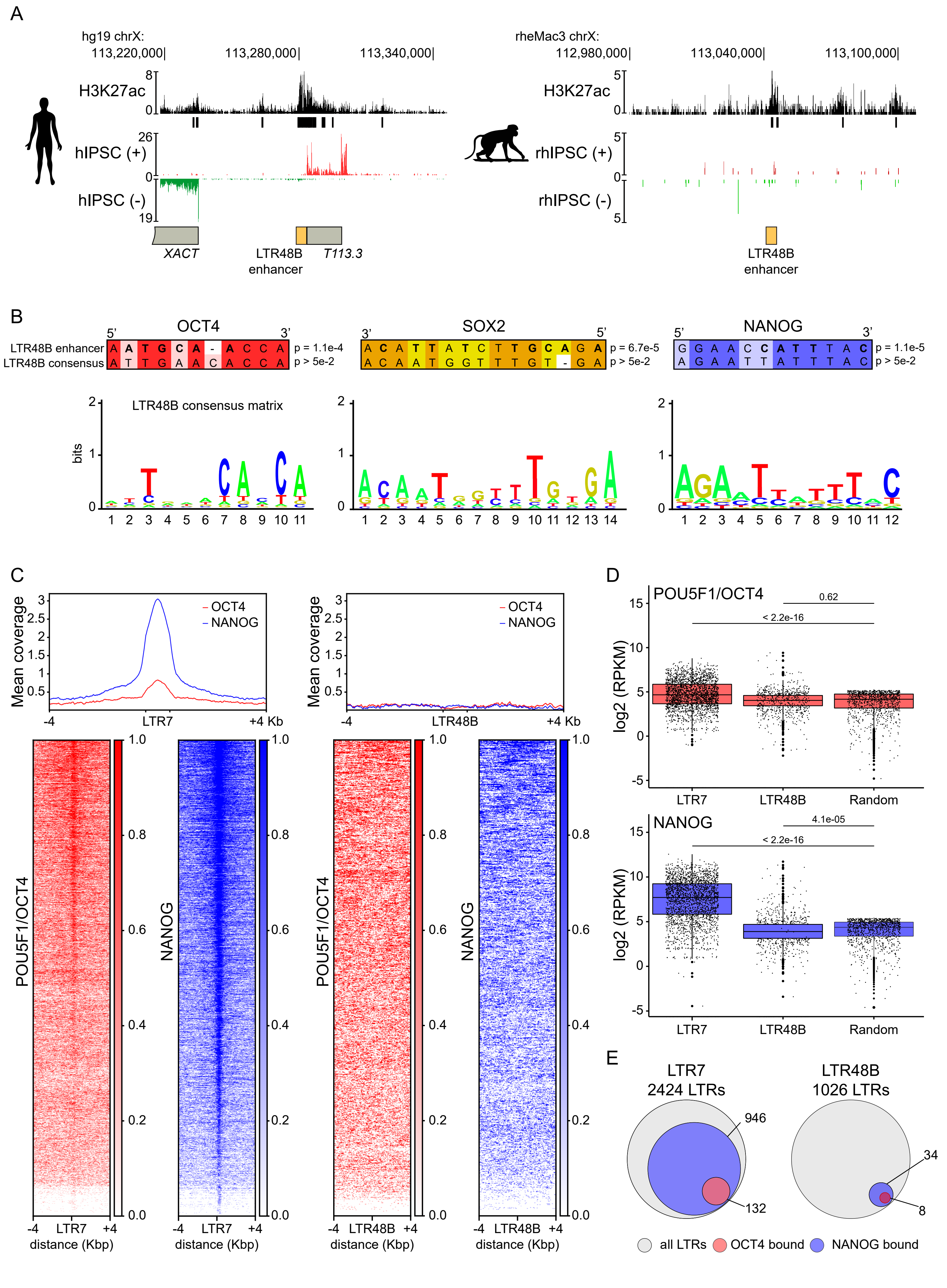

Supplementary Figure 6 – The LTR48B enhancer is active in species that do not have *XACT* and *T113.3* and evolved independently of the core of the LTR48B subfamily

A. ChIP-seq for H3K27ac and strand-specific RNA-seq from human (left panel) and rhesus macaque (right panel) iPSCs over a region spanning the *XACT* promoter and *T113.3* gene. Schematic representation of the *XACT* and *T113.3* genes as well as their common enhancer is shown below each panel. Dataset from[1].

B. Comparison between the *XACT*/*T113.3* LTR48B enhancer and all the other human LTR48B elements. Multiple alignment was performed between the LTR48B enhancer and the consensus sequence for the LTR48B family. P-values for the likelihood of the consensus sequence being a binding site for pluripotency transcription factors were determined using RSAT matrix-scan. The logo of all the human LTR48B sequences was obtained from the Dfam website[9].

C. Heatmap of log2-normalized ChIP-seq signal for OCT4 and NANOG in hESCs. The heatmaps show the distribution of the transcription factors in 8 kb windows (4 kb in each direction) centred at the middle of LTR7 (left panels) and LTR48B elements (right panels). The summary plot representing the average coverage of the transcription factors across the 8 kb windows around LTR7 (left) and LTR48B (right) are represented above the heatmaps. Dataset was from[10].

D. LTR7/HERVHs elements are enriched in OCT4 and NANOG pluripotency factors. Boxplot represents normalized number of reads (log2 reads per kilobase per million mapped reads [RPKM]) that mapped to individual LTR elements belonging to either LTR7 or LTR48B subfamilies. A thousand random regions across the genome were selected to use as a randomized control. p-values were calculated from a t-test. Dataset was from[10].

E. Number of LTR7 (left diagram) and LTR48B (right diagram) elements that are bound by OCT4 (red) and/or NANOG (blue) in hESCs.

Supplementary Table 1 – List of primers, gRNAs and siRNAs

qRT-PCR primers

	Forward	Reverse
GAPDH	AGCCACATCGCTCAGACAC	GCCCAATACGACCAAATCC
NANOG	CCAAAGGCAAACAACCCACTT	CGGGACCTTGTCTTCCTTTTT
OCT4	CGACCATCTGCCGCTTTG	GCCGCAGCTTACACATGTTCT
QXACT1	TCCCTCCCAGCAATGAAGGAAAGA	ATGGTGCAATGCCCACCAATAAGG
XACT Q1	CAGGAATGAGCAGGAGAGTTAAT	GTACATACGTGTAACCTGGTATCC
qXACT2	TGCCTCCCTCCTCGTGTTCAATAA	CCATGCCTTGCCTTATGCATCTCT
T113.3 FR2	CAGACGCCAAGCTTTAAGTAACTC	GCATTTGTAGTGAATAACAGATCGGA
T113.3 FR3	ATCACAGACGCCAAGCTTTA	CTACAGGGAGTGTGCTTGTG
HTR2C	GTGAACCTGAGGAATGCGGT	GGGCTCACAGAAATATCACATTG
GATA6	GAGGGTGAACCCGTGTGCAATG	TGGAAGTTGGAGTCATGGGAATGG
PAX6	GTCCATCTTTGCTTGGGAAA	TAGCCAGGTTGCGAAGAACT
ATRX	AATCACAGAAGCCGACAAGG	TGTGCAAGGAAGTCATGAAGC
NODAL	TGAGCCAACAAGAGGATCTG	TGGAAAATCTCAATGGCAAG
T113.3 int2	GGGTCACGAGGTTGATTGAT	GCTAGTTCCTGTCTTAGTCCATTAG
AMOT	CTGCTTGTTGGCAGTCTCTA	GATTCGGAGGATGCATGATTTC
HAND1	ATGGACGTGCTGGCCAAGGATG	TTAACTCCAGCCCAGACTTGC

ChIP qPCR primers

	Forward	Reverse
**XACT -2kb**	CCTGAGAAATCACAGGACACTAA	AAGTGGAATCTGTCTGCCTAAA
**XACT TSS**	TTCTGACACTTGCCTGTCTAAA	TATGGAGCCAAGAGCAAAGG
**XACT +2kb**	TCGTACTTGCTTGCTGATGTATAG	GGGAGGAAATATCATAATAACAAATGGG
**Between_25kb**	TTCCTTTCACATGGCAGTTCTA	GCTGAATGAGAGTCCATGTAAGT
**T113 -6kb**	CCACTGCAGGCACACTATAA	CTCTGAGTCTATGTGAACATCCC
**T113 -3kb**	GCTTTGTTTCTCAGATAGGTGTTC	GGCTTTCTGGGTCTGATAAGT
**T113 -2kb**	TTCACTTCTGCCAGCTTCTATT	TTCTGAGCCTCAACACAGTTTA
**T113 -2kb_2**	CTCCTGTTTCTCAGCCTTCTATC	TTTAAGGCAGGTGAGGGAATATC
**T113 TSS**	CGGGAAAGACTATCAGTTTGTGC	TCTGGATGTGTATGTGCAGGTC
**SOX2_TSS_FR**	GACAACTCCTGATACTTTTTTGAACAA	TGGTCGCTAGAAACCCATTTATTC
**B2M_FR**	GGCGCTCATTCTAGGACTTC	GGGCACCATTAGCAAGTCAC
**hXIC19_FR**	AAAAGGGAAAGTTAAGGGAAAAA	AAGAAAGGCAAAGTAGGCTTGA

Genotyping primers

**T113.3 DEL**	T113_reg1-3_F3	CCCTGGTGAAATAAGTTTGATGC
	T113_reg1-4_F1	GACAGAGAGAGACCCGGATAG
**T113.3 INV**	T113_INVdwn_F	GGAGAACTTTAACTATCAAGTAAGC
	T113_INVup4_R	TTTGTTGCCCTGTCACACTAC
**T113.3 WT**	T113_Insup_F	CAGTCTTTACAGCCTCTGATAGC
	T113_Insup_R	CTCACTACTTTCCTCATAAGTTCCA

**CTCF del**	T113_CTCF_F1	TTCACTCAACAACCCTGTCC
	T113_CTCF_R1	GCATTCCTCAGTCTTCCTTCTT
**CTCF Inv**	T113_CTCF_F1	TTCACTCAACAACCCTGTCC
	T113_IntCTCFs1	GCCTATCTCTGCCAAAGACTATC
**TFBS Del**	T113_TFBS_F1	CTGAACAGGATACCACCAAGAG

	T113_TFBS_R2	CTCTCAACCTCCAATCACGAG
**TFBS Inv**	T113_TFBS_F1	CTGAACAGGATACCACCAAGAG
	T113_IntTFBs1	CCTTCCTCTTGCTCACATCTC

Cloning Primers

	**Forward**	**Reverse**
**T113.3 Cloning**	GACTCAGCCTGCCTGCACCCAGGT	CTACAGGGAGTGTGCTTGTG

sgRNAs

**CRISPR KO**	
**T113.3**	
T113.3-sg_5'	GACAGCTATGTCCTAGATCT
T113.3-sg_3'	GCATGGCAGCAGGATCAGCA
**LTR48B enhancer**	
CTCF-sg_1	GATGGGCTGCACTGTCCGGTAGG
CTCF-sg_2	AGGCCCAAAATCTTAATGGTAGG
TFB-sg_3	GCATCAAACTTATTTCACCAGGG
TFB-sg_4	CTGACATAACTAATAGAAGCTGG

**CRISPR interference**	
**Sg CRISPRi XACT**	
sgi_X1	GTTACAGGTCACTCACTGTGA
sgi_X2	GTGGCACTGAGGTCTAGATTC
**sg CRISPRi T113.3**	
sgi_T1	GACAGCTATGTCCTAGATCT
sgi_T2	GATCGGGGCATAGGGATAAG

siRNA

	**Seller**	**Reference**
**POU5F1**	Ambion	S10872
**NANOG**	Ambion	s36650
**SOX2**	Dharmacon	L-011778-00-0005
**MALAT1**	Ambion	4390843
**Scrambled**	Ambion	4455877

Supplementary Table 2 – FISH probes

Origin	Reference
BACPAC	RP11-35D3
	a 10 kb fragment corresponding to *XIST* exon 1, gift from Dr. C. Brown, University of British Columbia
BACPAC	WI2-767I20

Supplementary Table 3 – Antibodies

ChIP antibodies						
	**Seller**	**Reference**	**Clone**	**Lot**	**Species**	**Ammount per IP**
**H3K4me3**	Millipore	04-745	MC315	2049822	Rabbit monoclonal	2 ml per IP
**H3K9me3**	Diagénode	pAb-193-050 (C15410193)	-	A1671-001P	Rabbit polyclonal	2 mg per IP
**H3K27ac**	Active motif	39133	-	-	Rabbit polyclonal	2 ml per IP

Western-blot antibodies			
	**Seller**	**Reference**	**Dilution**
**NANOG**	Abcam	ab21624	1:200
**SOX2**	Abcam	ab97959	1:1000
**OCT4**	Abcam	ab181557	1:1000
**TUBULIN**	Sigma Aldrich	T9026	1:10000
**H3.3**	Merck Millipore	09-838	1:1000

Supplementary Table 4 – Vectors

	**Source**
**pSpCas9(BB)-2A-GFP**	Addgene #48138
**pSpCas9(BB)-2A-mCherry**	in house
**pLKO5.sgRNA.EFS.tGFP**	Addgene #57823
**pMD2.G**	Addgene #12259
**psPAX2**	Addgene #12260
**dCas9/KRAB-mCherry construct**	Ref 11

Supplementary References

1. Bujold, D. *et al.* The International Human Epigenome Consortium Data Portal. *Cell Systems* **3**, 496-499.e2 (2016).
2. Wang, J. *et al.* Primate-specific endogenous retrovirus-driven transcription defines naive-like stem cells. *Nature* **516**, 405–409 (2014).
3. Blakeley, P. *et al.* Defining the three cell lineages of the human blastocyst by single-cell RNA-seq. *Development* **142**, 3151–3165 (2015).
4. Xue, Z. *et al.* Genetic programs in human and mouse early embryos revealed by single-cell RNA sequencing. *Nature* **500**, 593–597 (2013).
5. Yan, L. *et al.* Single-cell RNA-Seq profiling of human preimplantation embryos and embryonic stem cells. *Nature Structural & Molecular Biology* **20**, 1131–1139 (2013).
6. Petropoulos, S. *et al.* Single-Cell RNA-Seq Reveals Lineage and X Chromosome Dynamics in Human Preimplantation Embryos. *Cell* **165**, 1012–1026 (2016).
7. The ENCODE Project Consortium. An integrated encyclopedia of DNA elements in the human genome. *Nature* **489**, 57–74 (2012).
8. Zheng, R. *et al.* Cistrome Data Browser: expanded datasets and new tools for gene regulatory analysis. *Nucleic Acids Res* **47**, D729–D735 (2019).
9. Hubley, R. *et al.* The Dfam database of repetitive DNA families. *Nucleic Acids Res* **44**, D81–D89 (2016).
10. Lyu, X., Rowley, M. J. & Corces, V. G. Architectural Proteins and Pluripotency Factors Cooperate to Orchestrate the Transcriptional Response of hESCs to Temperature Stress. *Molecular Cell* **71**, 940-955.e7 (2018).
11. Furlan, G. *et al*. The Ftx noncoding locus controls X chromosome inactivation independently of its RNA products. *Molecular Cell* **70,** 462-472 (2018).
