# Supplementary material for: A primate-specific retroviral enhancer wires the XACT lncRNA into the core pluripotency network in humans
Source: Nat Commun. 2019 Dec 11;10:5652. doi: 10.1038/s41467-019-13551-1 (PMC6906429; doi:10.1038/s41467-019-13551-1)
Supplement: Supplementary file 3 — Reporting Summary [file 41467_2019_13551_MOESM3_ESM.pdf]

nature research

Corresponding author(s): Claire Rougeulle, Miguel Casanova

Last updated by author(s): 2019/09/23

Reporting Summary

Nature Research wishes to improve the reproducibility of the work that we publish. This form provides structure for consistency and transparency in reporting. For further information on Nature Research policies, see Authors & Referees and the Editorial Policy Checklist.

Statistics

For all statistical analyses, confirm that the following items are present in the figure legend, table legend, main text, or Methods section.

n/a	Confirmed	
☐	☒	The exact sample size (n) for each experimental group/condition, given as a discrete number and unit of measurement
☐	☒	A statement on whether measurements were taken from distinct samples or whether the same sample was measured repeatedly
☐	☒	The statistical test(s) used AND whether they are one- or two-sided<br>*Only common tests should be described solely by name; describe more complex techniques in the Methods section.*
☒	☐	A description of all covariates tested
☒	☐	A description of any assumptions or corrections, such as tests of normality and adjustment for multiple comparisons
☐	☒	A full description of the statistical parameters including central tendency (e.g. means) or other basic estimates (e.g. regression coefficient) AND variation (e.g. standard deviation) or associated estimates of uncertainty (e.g. confidence intervals)
☐	☒	For null hypothesis testing, the test statistic (e.g. F, t, r) with confidence intervals, effect sizes, degrees of freedom and P value noted<br>*Give P values as exact values whenever suitable.*
☒	☐	For Bayesian analysis, information on the choice of priors and Markov chain Monte Carlo settings
☒	☐	For hierarchical and complex designs, identification of the appropriate level for tests and full reporting of outcomes
☐	☒	Estimates of effect sizes (e.g. Cohen's d, Pearson's r), indicating how they were calculated

Our web collection on statistics for biologists contains articles on many of the points above.

Software and code

Policy information about availability of computer code

Data collection	All fluorescent microscopy images were taken on a fluorescence DMI-6000 inverted microscope with a motorized stage (Leica), equipped with a CCD Camera HQ2 (Roper Scientifics) and a HCX PL APO 100X oil objective (numerical aperture, 1.4, Leica) using the Metamorph software (version 7.04,Roper Scientifics).<br>All qPCR results were collected on a ViiA-7 real-time thermal cycler (Applied Biosystems).<br>ChIP-seq and RNA-seq datasets were collected using fastq-dump (v2.9.6)<br>Jaspar and Transfac were used to get the binding motif matrices.<br>LTR7 and LTR48B consensus sequences and matrices were collected using the Dfam website.

Data analysis	All analysis of the data presented are extensively described in the material & method section of the paper.<br>For RNA-seq analysis, RPKM (Reads Per Kilobase Millions) tables of single-cell datasets performed on human embryos (Petropoulos et al., 2016, Blakeley et al., 2015, Xue et al., 2013 and Yan et al., 2013)) were obtained from a previous analysis (Vallot et al., 2017). All graphical plots were obtained using R (version 3.0.2) with the ggplot2 package (version 1.0.1).<br>For ChIP-seq analysis, reads were aligned with bowtie2 (v2.3.4.3), filtered and sorted with samtools (v1.9). Uniquely mapped reads were selected with get_unique_reads.pl (Rica, L. et al. Genome Biology, 2016). Picard (v2.20.1) was used to remove PCR duplicates and deeptools (v3.3.0) to create binding profiles for each studied LTR. ChIP-seq peaks were called with macs2 (v2.1.2).<br>Bedtools (v2.28.0) was used to cross LTRs positions and called peaks.<br>Hi-C datasets heatmaps (Bonev et al., 2017; Dekker et al., 2017; Rao et al., 2014) represents raw observed matrix visualized at a 5kb resolution and were visualized using the Juicebox suite (Durand et al., 2016a). For ChiA-PET datasets, long-range chromatin interactions and signals tracks were obtained from: (i) the ENCODE project (https://www.encodeproject.org) for CTCF (ENCSR000CAC) (Li et al., 2012). All data were visualized with the Integrative Genomics Viewer (Robinson et al., 2011) or the UCSC Genome browser (Kent et al., 2002).<br>Blast (v2.9.0) and UCSC liftover (v377) were used to discover homologous regions between species. MAFFT (v7.407) allowed to align homologous sequences and calculate conservations.<br>RSAT Metazoa website was used to compare binding motifs matrices.<br>For other analysis, Graphpad Prism (Statistics), Excel, Image J (stack processing & analysis) were used.

For manuscripts utilizing custom algorithms or software that are central to the research but not yet described in published literature, software must be made available to editors/reviewers. We strongly encourage code deposition in a community repository (e.g. GitHub). See the Nature Research guidelines for submitting code & software for further information.

Data

Policy information about availability of data

All manuscripts must include a data availability statement. This statement should provide the following information, where applicable:

- Accession codes, unique identifiers, or web links for publicly available datasets
- A list of figures that have associated raw data
- A description of any restrictions on data availability

All data generated or analyzed during this study are included in the published article (and its supplementary information).

Field-specific reporting

Please select the one below that is the best fit for your research. If you are not sure, read the appropriate sections before making your selection.

☒ Life sciences ☐ Behavioural & social sciences ☐ Ecological, evolutionary & environmental sciences

For a reference copy of the document with all sections, see nature.com/documents/nr-reporting-summary-flat.pdf

Life sciences study design

All studies must disclose on these points even when the disclosure is negative.

Sample size	No sample size pre-determination was performed. The sample size were chosen to ensure the findings could be reproduced by performing multiple independent experiments (n>= 3).
Data exclusions	No data were excluded from any analysis
Replication	Experiments presented in this manuscript were reproduced. All replication attempts were successful.
Randomization	This is not relevant in this study
Blinding	Blinding was not relevant in this study

Reporting for specific materials, systems and methods

We require information from authors about some types of materials, experimental systems and methods used in many studies. Here, indicate whether each material, system or method listed is relevant to your study. If you are not sure if a list item applies to your research, read the appropriate section before selecting a response.

Materials & experimental systems

n/a	Involved in the study
☐	☒ Antibodies
☐	☒ Eukaryotic cell lines
☒	☐ Palaeontology
☒	☐ Animals and other organisms
☒	☐ Human research participants
☒	☐ Clinical data

Methods

n/a	Involved in the study
☒	☐ ChIP-seq
☒	☐ Flow cytometry
☒	☐ MRI-based neuroimaging

Antibodies

Antibodies used

This has been reported in the methods specific sections.

ChIP:H3K9me3 Diagenode, Cat# pAb-193-050, Lot: A1671-001P; H3K4me3 Millipore Cat# 04-745, Lot: 2049822; H3K27ac Active Motif,Cat# 39133

Western blot: NANOG Abcam, Cat#: ab21624; SOX2 Abcam, Cat#: ab97959; OCT4 Abcam, Cat#: ab181557; NANOG Sigma Aldrich Cat#: T9026; H3.3 Millipore, Cat#: 09-838

Validation

These antibodies have been validated by the providers and by the community (previous publications) using ChIP and ChIP-seq experiments. IF and Western antibodies have also been validated and extensively used in previous publications.

Eukaryotic cell lines

Policy information about cell lines

Cell line source(s)

H1 and H9: WiCell RESEARCH INSTITUTE
WIBR2: Whitehead Institute for Biomedical Research (WIBR)

Authentication

The cells lines have been authenticated by the providers.

Mycoplasma contamination

The cells were tested for mycoplasma contamination on a regular basis.

Commonly misidentified lines
(See ICLAC register)

No cell lines used in this paper listed in the database of misidentified cell lines maintained by ICLAC.
