# Supplementary material for: A primate-specific retroviral enhancer wires the XACT lncRNA into the core pluripotency network in humans
Source: Nat Commun. 2019 Dec 11;10:5652. doi: 10.1038/s41467-019-13551-1 (PMC6906429; doi:10.1038/s41467-019-13551-1)
Supplement: Supplementary file 4 — Source Data [file 41467_2019_13551_MOESM4_ESM.docx]

**Figure 2**

- Figure 2 B

|  | XACT | | | T113.3 | | |
| --- | --- | --- | --- | --- | --- | --- |
|  | n1 | n2 | n3 | n1 | n2 | n3 |
| D0 | 0,00124 | 0,0019 | 0,000749 | 0,00564 | 0,00557 | 0,00298 |
| D1 | 0,00237 | 0,00233 |  | 0,00471 | 0,0045 |  |
| D2 | 0,00242 | 0,00221 | 0,00147 | 0,00466 | 0,00489 | 0,00581 |
| D3 | 0,00225 | 0,00264 | 0,00258 | 0,00487 | 0,00352 | 0,00533 |
| D4 | 0,000932 | 0,000929 | 0,000824 | 0,00314 | 0,00231 | 0,00204 |
| D5 | 0,000385 | 0,000318 | 0,000397 | 0,00213 | 0,00143 | 0,000865 |
| D6 | 0,000184 | 0,000131 | 0,000209 | 0,00121 | 0,000653 | 0,000636 |
| D7 | 0,000119 | 0,0000653 | 0,0000342 | 0,000317 | 0,00021 | 0,000138 |
| D8 | 0,0000544 | 0,0000355 | 0,000239 | 0,0002 | 0,00015 | 0,000799 |
| D9 | 0,0000422 | 0,0000597 | 0,0000675 | 0,000151 | 0,000221 | 0,000256 |
| D10 | 4,31E-06 | 0,000011 |  | 0,0000473 | 0,0000103 |  |

**Supp Figure 2**

- Supp Figure 2 B

|  | XACT | | T113.3 | |
| --- | --- | --- | --- | --- |
|  | n1 | n2 | n1 | n2 |
| D0 | 0,001344 | 0,0016459 | 0,0023276 | 0,0008812 |
| D1 | 0,0047436 | 0,0019973 | 0,0037538 | 0,0013225 |
| D2 | 0,0027902 | 0,0081208 | 0,0037223 | 0,0019998 |
| D3 | 0,0051058 | 0,0085802 | 0,0043108 | 0,003116 |
| D4 | 0,0009308 | 0,006513 | 0,0026368 | 0,0024846 |
| D5 | 0,0013007 | 0,003785 | 0,0022815 | 0,0012064 |
| D6 | 0,0011871 | 0,0030591 | 0,0015355 | 0,0012587 |
| D7 | 0,0004853 | 0,0003629 | 0,0011157 | 0,0002134 |
| D8 | 0,000241 | 0,000873 | 0,0004773 | 0,0005963 |
| D9 | 0,0001137 | 0,0001457 | 0,0002787 | 5,9E-05 |
| D10 | 0,0001065 | 0,0001745 | 0,0001403 | 9,984E-05 |

**Figure 3**

- Figure 3 A

*XACT* CRISPRi

|  | *XACT* expression | | | |
| --- | --- | --- | --- | --- |
|  | n1 | n2 | n3 | n4 |
| no_sg | 1 | 1 | 1 |  |
| sg_empty | 1,3651 | 0,9088307 | 0,9482917 |  |
| sgi_X1 | 0,2707509 | 0,1880614 | 0,1513823 | 0,1678665 |
| sgi_X2 | 0,4214288 | 0,2249814 | 0,3361028 | 0,1744153 |

|  | *T113*.3 expression | | | |
| --- | --- | --- | --- | --- |
|  | n1 | n2 | n3 | n4 |
| no_sg | 1 | 1 | 1 |  |
| sg_empty | 1,114516 | 1,348041 | 0,978356 |  |
| sgi_X1 | 1,076323 | 1,090168 | 1,293663 | 0,8653142 |
| sgi_X2 | 1,17218 | 1,074705 | 1,145288 | 0,9824013 |

| *T113.3* CRISPRi |  |  |  |  |
| --- | --- | --- | --- | --- |
|  | *XACT* expression | | | |
|  | n1 | n2 | n3 | n4 |
| no_sg | 1 | 1 | 1 |  |
| sg_empty | 1,3651 | 0,9088307 | 0,9482917 |  |
| sgi_T1 | 0,9535 | 0,5069826 | 0,1888289 | 0,2148456 |
| sgi_T2 | 0,64684 | 0,2842075 | 0,3272831 | 0,3109128 |

|  | *T113.3* expression | | | |
| --- | --- | --- | --- | --- |
|  | n1 | n2 | n3 | n4 |
| no_sg | 1 | 1 | 1 |  |
| sg_empty | 1,114516 | 1,348041 | 0,978356 |  |
| sgi_T1 | 0,1361561 | 0,132702 | 0,0903017 | 0,0578805 |
| sgi_T2 | 0,1262136 | 0,117741 | 0,0726065 | 0,0649616 |

- Figure 3B

H3K9me3

|  | sgT113 | | | | | |
| --- | --- | --- | --- | --- | --- | --- |
| -2kb | 1,067444 | 1,091958 | 1,007456 | 1,268663 | 0,9241849 | 0,633494 |
| XACT TSS | 0,936883 | 1,17718 | 1,094038 | 1,106642 | 0,8135393 | 0,433687 |
| +2kb | 1,321844 | 0,891986 | 1,206789 | 1,434784 | 1,162386 | 0,788624 |
|  |  |  |  |  |  |  |
| +/- 25kb | 2,468193 | 3,089053 | 2,969659 | 2,635407 | 2,346227 | 1,708583 |
|  |  |  |  |  |  |  |
| -6kb | 7,202165 | 5,815594 | 7,65542 | 6,700153 | 6,309668 | 5,239777 |
| -3kb (CTCF) | 4,523253 | 3,459345 | 3,976531 | 5,143985 | 3,649905 | 2,212374 |
| -2kb (TFBS) | 4,603053 | 4,243932 | 3,981018 | 5,485734 | 4,039405 | 2,667946 |
| T113.3 TSS | 8,745668 | 10,80078 | 12,71046 | 15,91645 | 17,6436 | 12,40107 |

H3K9me3

|  | sgXACT | | | | | |
| --- | --- | --- | --- | --- | --- | --- |
| -2kb | 2,592589 | 2,635835 | 4,673979 | 3,393391 | 3,34462 | 3,443474 |
| XACT TSS | 6,632897 | 8,172054 | 13,96526 | 9,073084 | 9,998083 | 10,75768 |
| +2kb | 6,514676 | 6,053081 | 10,7455 | 2,773123 | 2,819385 | 3,966843 |
|  |  |  |  |  |  |  |
| +/- 25kb | 1,924318 | 2,042152 | 2,855694 | 1,998195 | 2,060621 | 1,8248 |
|  |  |  |  |  |  |  |
| -6kb | 2,706077 | 2,9292 | 5,372204 | 3,510415 | 3,08319 | 3,341698 |
| -3kb (CTCF) | 1,030861 | 0,708158 | 0,700866 | 0,870635 | 0,828548 | 0,618985 |
| -2kb (TFBS) | 0,761164 | 0,568015 | 1,239709 | 0,736283 | 0,585443 | 0,588871 |
| T113.3 TSS | 0,994009 | 1,059545 | 1,966398 | 1,087167 | 1,51754 | 1,465749 |

H3K9me3

|  | sgEmpty | | |
| --- | --- | --- | --- |
| -2kb | 0,9348861 | 0,911018 | 0,942694 |
| XACT TSS | 0,8117644 | 0,695201 | 0,861655 |
| +2kb | 0,9681717 | 0,685878 | 0,896456 |
|  |  |  |  |
| +/- 25kb | 1,533505 | 1,440644 | 1,434593 |
|  |  |  |  |
| -6kb | 2,684966 | 2,333433 | 2,512375 |
| -3kb (CTCF) | 0,7405062 | 0,650048 | 0,588735 |
| -2kb (TFBS) | 0,6194882 | 0,37532 | 0,690427 |
| T113.3 TSS | 1,152337 | 1,330934 | 0,911874 |

H3K9me3

|  | No sg | | |
| --- | --- | --- | --- |
| -2kb | 0,953595 | 0,95931 | 1,080636 |
| XACT TSS | 0,766374 | 0,946628 | 1,233513 |
| +2kb | 1,074124 | 0,998716 | 1,522294 |
|  |  |  |  |
| +/- 25kb | 1,740743 | 1,500617 | 2,224187 |
|  |  |  |  |
| -6kb | 2,619552 | 2,358403 | 3,152037 |
| -3kb (CTCF) | 0,751152 | 0,700991 | 0,660317 |
| -2kb (TFBS) | 0,775145 | 0,427289 | 0,48997 |
| T113.3 TSS | 1,190821 | 1,19777 | 1,470296 |

H3K9me3

|  | sgT113 | | | | | |
| --- | --- | --- | --- | --- | --- | --- |
| SOX2_FR | 0,323688 | 0,401978 | 0,241507 | 0,296029 | 0,3678093 | 0,117128 |
| hXIC19_FR | 7,700387 | 9,134478 | 9,926591 | 9,027365 | 6,804445 | 5,45181 |

H3K9me3

|  | sgXACT | | | | | |
| --- | --- | --- | --- | --- | --- | --- |
| SOX2_FR | 0,324583 | 0,292921 | 0,35458 | 0,330799 | 0,399445 | 0,247384 |
| hXIC19_FR | 7,225149 | 7,052647 | 13,51337 | 8,447473 | 8,111121 | 8,241167 |

H3K9me3

|  | sgEmpty | | |
| --- | --- | --- | --- |
| SOX2_FR | 0,2245621 | 0,361656 | 0,237169 |
| hXIC19_FR | 6,907511 | 7,953135 | 8,061175 |

H3K9me3

|  | No sg | | |
| --- | --- | --- | --- |
| SOX2_FR | 0,318923 | 0,355006 | 0,458461 |
| hXIC19_FR | 8,09898 | 7,09112 | 9,896479 |

**Supp figure 3**

- Supp Figure 3 B

*XACT* CRISPRi

|  | NANOG expression | | | | OCT4 expression | | |
| --- | --- | --- | --- | --- | --- | --- | --- |
|  | n1 | n2 | n3 | n4 | n1 | n2 | n3 |
| no_sg | 1 | 1 | 1 |  | 1 | 1 | 1 |
| sg_empty | 1,242531 | 0,9027258 | 1,042239 |  | 0,8583797 | 0,8957396 |  |
| sgi_X1 | 1,187178 | 0,6983034 | 0,7710758 | 0,8925083 | 0,9360103 | 0,9322079 | 0,9318567 |
| sgi_X2 | 1,214801 | 0,7256923 | 1,032981 | 0,7285459 | 0,762911 | 1,013681 | 0,8290195 |

*T113*.3 CRISPRi

|  | NANOG expression | | | | OCT4 expression | | |
| --- | --- | --- | --- | --- | --- | --- | --- |
|  | n1 | n2 | n3 | n4 | n1 | n2 | n3 |
| no_sg | 1 | 1 | 1 |  | 1 | 1 | 1 |
| sg_empty | 1,242531 | 0,9027258 | 1,042239 |  | 0,8583797 | 0,8957396 |  |
| sgi_T1 | 1,121502 | 0,8556944 | 0,642377 | 0,5754447 | 0,8628045 | 1,755723 | 0,7466552 |
| sgi_T2 | 0,9446941 | 0,7884026 | 0,7462629 | 0,8193058 | 0,9166798 | 0,9716139 | 0,9296448 |

- Supp Figure 3 C

H3K4me3

|  | sgT113.3 | | | | | |
| --- | --- | --- | --- | --- | --- | --- |
| -2kb | 1,191829 | 1,053441 | 0,986163 | 0,854026 | 1,043633 | 0,661286 |
| TSS | 7,025743 | 5,978172 | 7,636164 | 6,42755 | 5,778958 | 4,211614 |
| +2kb | 0,535984 | 0,401797 | 0,314003 | 0,417539 | 0,368911 | 0,309657 |
|  |  |  |  |  |  |  |
| +25kb | 0,667118 | 0,286223 | 0,249884 | 0,301203 | 0,258889 | 0,227045 |
|  |  |  |  |  |  |  |
| -6kb | 0,920179 | 0,739504 | 0,745523 | 0,796263 | 0,645939 | 0,601968 |
| -3kb (CTCF) | 1,003031 | 0,685032 | 0,701143 | 0,829131 | 0,685466 | 0,48561 |
| -2kb (TFBS) | 1,102166 | 0,897237 | 0,726351 | 0,971394 | 0,76403 | 0,611114 |
| TSS | 1,695229 | 1,41904 | 1,371031 | 2,21657 | 2,13364 | 1,799442 |

H3K4me3

|  | sgXACT | | | | | |
| --- | --- | --- | --- | --- | --- | --- |
| -2kb | 1,087447 | 0,653392 | 0,616677 | 0,490157 | 0,586896 | 0,197014 |
| TSS | 3,835085 | 2,561595 | 2,874319 | 2,269843 | 2,201876 | 0,996368 |
| +2kb | 1,714772 | 0,762663 | 0,776523 | 0,304623 | 0,357599 | 0,180441 |
|  |  |  |  |  |  |  |
| +25kb | 0,649541 | 0,318675 | 0,270116 | 0,149925 | 0,171986 | 0,083278 |
|  |  |  |  |  |  |  |
| -6kb | 0,98172 | 0,614846 | 0,482868 | 0,390061 | 0,301407 | 0,155495 |
| -3kb (CTCF) | 1,20199 | 0,842616 | 0,708353 | 0,446238 | 0,505346 | 0,166232 |
| -2kb (TFBS) | 1,678592 | 1,242401 | 1,079724 | 0,518077 | 0,726965 | 0,281594 |
| TSS | 6,51366 | 3,860572 | 2,733986 | 1,992629 | 2,401813 | 0,666146 |

H3K4me3

|  | sgEmpty | | |
| --- | --- | --- | --- |
| -2kb | 1,571427 | 1,008096 | 2,084286 |
| TSS | 10,26759 | 3,825176 | 13,79486 |
| +2kb | 0,677671 | 0,341394 | 0,579467 |
|  |  |  |  |
| +25kb | 0,335192 | 0,096677 | 0,167331 |
|  |  |  |  |
| -6kb | 0,674877 | 0,150168 | 0,436541 |
| -3kb (CTCF) | 0,612154 | 0,26894 | 0,468156 |
| -2kb (TFBS) | 0,953282 | 0,35074 | 0,834538 |
| TSS | 3,220989 | 1,288576 | 1,807845 |

H3K4me3

|  | No sg | | |
| --- | --- | --- | --- |
| -2kb | 2,481274 | 2,311602 | 2,065304 |
| TSS | 17,24184 | 13,24618 | 14,7199 |
| +2kb | 0,96126 | 0,657585 | 0,671899 |
|  |  |  |  |
| +25kb | 0,444366 | 0,252017 | 0,280517 |
|  |  |  |  |
| -6kb | 0,627088 | 0,540558 | 0,432241 |
| -3kb (CTCF) | 0,751541 | 0,899726 | 0,559216 |
| -2kb (TFBS) | 1,398602 | 1,262343 | 1,029007 |
| TSS | 4,606955 | 3,577352 | 2,249564 |

H3K4me3

|  | sgT113.3 | | | | | |
| --- | --- | --- | --- | --- | --- | --- |
| SOX2_TSS_FR | 17,0789 | 12,90409 | 10,37147 | 14,23394 | 13,74883 | 7,40192 |
| B2M_FR | 21,2963 | 18,26975 | 26,09149 | 19,30612 | 22,24496 | 18,13048 |
| hXIC19_FR | 1,210525 | 0,74676 | 0,74157 | 1,085745 | 0,877699 | 0,66357 |

H3K4me3

|  | sgXACT | | | | | |
| --- | --- | --- | --- | --- | --- | --- |
| SOX2_TSS_FR | 22,21683 | 13,9353 | 10,68903 | 8,663421 | 9,187248 | 2,17202 |
| B2M_FR | 31,15924 | 22,74619 | 25,29091 | 14,12369 | 13,95518 | 6,029264 |
| hXIC19_FR | 1,638637 | 0,978664 | 0,997108 | 0,549615 | 0,685802 | 0,224427 |

H3K4me3

|  | sgEmpty | | |
| --- | --- | --- | --- |
| SOX2_TSS_FR | 11,99287 | 3,251753 | 8,524537 |
| B2M_FR | 15,69527 | 5,61141 | 22,60565 |
| hXIC19_FR | 0,957463 | 0,313481 | 0,691654 |

H3K4me3

|  | No sg | | |
| --- | --- | --- | --- |
| SOX2_TSS_FR | 18,11362 | 11,94194 | 10,28075 |
| B2M_FR | 25,37703 | 19,62534 | 26,155 |
| hXIC19_FR | 1,220519 | 0,746907 | 0,934556 |

H3K27ac

|  | sgT113.3 | | | | | |
| --- | --- | --- | --- | --- | --- | --- |
| -2kb | 6,6368 | 4,627282 | 5,247437 | 4,475303 | 4,916093 | 5,391608 |
| TSS | 5,540172 | 4,08208 | 4,761806 | 4,196896 | 4,615222 | 5,241918 |
| +2kb | 3,772971 | 2,62324 | 2,956485 | 3,10092 | 2,759803 | 3,087306 |
|  |  |  |  |  |  |  |
| +25kb | 1,76069 | 1,114571 | 1,21357 | 1,316368 | 1,203003 | 1,300168 |
|  |  |  |  |  |  |  |
| -6kb | 2,761117 | 1,655003 | 2,219171 | 1,935967 | 1,819842 | 2,172503 |
| -3kb (CTCF) | 7,635578 | 5,509953 | 5,303519 | 6,321951 | 5,971229 | 4,781646 |
| -2kb (TFBS) | 9,010863 | 5,831084 | 7,234832 | 6,220118 | 6,906699 | 7,952558 |
| TSS | 3,906849 | 3,154922 | 3,775401 | 3,126351 | 3,52397 | 3,690255 |

H3K27ac

|  | sgXACT | | | | | |
| --- | --- | --- | --- | --- | --- | --- |
| -2kb | 5,025654 | 4,24474 | 4,039769 | 4,768181 | 3,62602 | 3,986891 |
| TSS | 3,375917 | 2,403932 | 2,533911 | 2,714358 | 2,326256 | 2,617102 |
| +2kb | 2,880729 | 2,135122 | 1,71334 | 2,544634 | 2,112622 | 2,492113 |
|  |  |  |  |  |  |  |
| +25kb | 1,978919 | 1,569367 | 1,213664 | 1,465164 | 1,152965 | 1,482047 |
|  |  |  |  |  |  |  |
| -6kb | 3,876821 | 2,804548 | 2,210451 | 2,625204 | 1,813629 | 2,030788 |
| -3kb (CTCF) | 9,824064 | 7,760636 | 5,670415 | 8,141461 | 5,666503 | 5,281032 |
| -2kb (TFBS) | 10,74104 | 8,505624 | 7,865749 | 10,64517 | 6,966224 | 8,486518 |
| TSS | 7,890454 | 5,511177 | 4,887714 | 6,741782 | 4,654266 | 5,000126 |

H3K27ac

|  | sgEmpty | | |
| --- | --- | --- | --- |
| -2kb | 8,347986 | 6,768324 | 6,173821 |
| TSS | 7,33645 | 5,810084 | 6,123608 |
| +2kb | 4,740988 | 3,502437 | 3,524622 |
|  |  |  |  |
| +25kb | 1,930567 | 1,475526 | 1,302636 |
|  |  |  |  |
| -6kb | 3,321372 | 2,554652 | 2,277472 |
| -3kb (CTCF) | 9,309984 | 7,634892 | 5,496588 |
| -2kb (TFBS) | 14,66912 | 10,8565 | 9,697486 |
| TSS | 8,703107 | 6,472028 | 5,361221 |

H3K27ac

|  | No sg | | |
| --- | --- | --- | --- |
| -2kb | 6,549219 | 6,660806 | 7,15459 |
| TSS | 5,738872 | 5,988273 | 5,863436 |
| +2kb | 3,951773 | 3,850056 | 3,581299 |
|  |  |  |  |
| +25kb | 1,433715 | 1,609594 | 1,415225 |
|  |  |  |  |
| -6kb | 2,699627 | 2,756696 | 2,407052 |
| -3kb (CTCF) | 7,983349 | 7,598429 | 6,701473 |
| -2kb (TFBS) | 11,95777 | 11,04783 | 12,20285 |
| TSS | 7,693804 | 7,076501 | 5,9044 |

H3K27ac

|  | sgT113.3 | | | | | |
| --- | --- | --- | --- | --- | --- | --- |
| SOX2_TSS_FR | 2,407689 | 1,533406 | 0,693583 | 1,818479 | 1,393002 | 0,651406 |
| B2M_FR | 2,778933 | 2,094387 | 2,677639 | 2,223191 | 2,151136 | 2,37651 |
| hXIC19_FR | 1,248766 | 0,774902 | 1,000557 | 1,060644 | 0,794763 | 0,880473 |

H3K27ac

|  | sgXACT | | | | | |
| --- | --- | --- | --- | --- | --- | --- |
| SOX2_TSS_FR | 2,71925 | 1,132194 | 0,656217 | 2,138752 | 1,181814 | 0,729634 |
| B2M_FR | 3,548411 | 2,188361 | 1,994331 | 2,968866 | 1,730526 | 1,830566 |
| hXIC19_FR | 1,419748 | 0,813182 | 0,909343 | 0,969021 | 0,536305 | 0,977284 |

H3K27ac

|  | sgEmpty | | |
| --- | --- | --- | --- |
| SOX2_TSS_FR | 2,716239 | 1,386446 | 0,769172 |
| B2M_FR | 3,560131 | 2,450723 | 2,343335 |
| hXIC19_FR | 1,257979 | 0,775604 | 0,918263 |

H3K27ac

|  | No sg | | |
| --- | --- | --- | --- |
| SOX2_TSS_FR | 2,100902 | 1,479253 | 0,920927 |
| B2M_FR | 2,78976 | 2,517745 | 2,428205 |
| hXIC19_FR | 1,136211 | 0,813716 | 0,848891 |

**Figure 4**

- Figure 4A

| *T113*.3 expression | no LNA | LNA_SCR | LNA_T1 | LNA_T2 |
| --- | --- | --- | --- | --- |
| n=1 | 1 | 1,402012 | 0,4291337 | 0,1230229 |
| n=2 | 1 | 1,5632 | 0,2850923 | 0,1110285 |
| n=3 | 1 | 0,8461586 | 0,3852856 |  |
| n=4 | 1 | 1,008352 | 0,196827 | 0,1415124 |
| n=5 | 1 | 1,103791 | 0,2528373 | 0,1924206 |
| n=6 | 1 | 0,8490452 | 0,4098812 | 0,2628093 |
| n=7 | 1 | 1,542209 | 0,4123665 | 0,1149436 |

| *XACT* expression | no LNA | LNA_SCR | LNA_T1 | LNA_T2 |
| --- | --- | --- | --- | --- |
| n=1 | 1 | 1,053361 | 1,153086 | 1,229012 |
| n=2 | 1 | 0,9378963 | 1,388955 | 0,7573332 |
| n=3 | 1 | 2,061937 | 1,291457 |  |
| n=4 | 1 | 1,815038 | 1,212513 | 1,350038 |
| n=5 | 1 | 0,7463207 | 1,529882 | 0,8909792 |
| n=6 | 1 | 0,9933885 | 0,9301139 | 1,163225 |
| n=7 | 1 | 0,9221027 | 0,6256812 | 0,7573326 |

| *T113*.3 expression | no LNA | LNA_SCR | LNA_X1 | LNA_X2 |
| --- | --- | --- | --- | --- |
| n=1 | 1 | 1,402012 | 1,469678 | 1,460031 |
| n=2 | 1 | 1,5632 | 1,199557 | 1,03419 |
| n=3 | 1 | 0,8461586 | 0,8876113 | 1,033114 |
| n=4 | 1 | 1,008352 | 0,8790396 | 0,7290047 |
| n=5 | 1 | 1,103791 | 1,176955 | 0,7361702 |
| n=6 | 1 | 0,8490452 | 0,8702774 | 0,7217359 |
| n=7 | 1 | 1,542209 | 0,8645366 | 1,074749 |

| *XACT* expression | no LNA | LNA_SCR | LNA_X1 | LNA_X2 |
| --- | --- | --- | --- | --- |
| n=1 | 1 | 1,053361 | 0,1273173 | 0,0558229 |
| n=2 | 1 | 0,9378963 | 0,1389367 | 0,0334118 |
| n=3 | 1 | 2,061937 | 0,510506 | 0,1558252 |
| n=4 | 1 | 1,815038 | 0,2750947 | 0,1915775 |
| n=5 | 1 | 0,7463207 | 0,1304607 | 0,1468571 |
| n=6 | 1 | 0,9933885 | 0,212159 | 0,1189174 |
| n=7 | 1 | 0,9221027 | 0,1390331 | 0,0332962 |

- Figure 4B

| *T113*.3 expression | WT | KO | INV |
| --- | --- | --- | --- |
|  | 0,003698 | 3,747E-06 | 1,584E-05 |
|  | 0,0043156 | 9,55E-06 | 1,581E-05 |
|  | 0,0050759 | 1,06E-05 | 3,344E-05 |

| T113.3 int2 expression | WT | KO | INV |
| --- | --- | --- | --- |
|  | 0,0005205 | 2,034E-06 | 3,839E-07 |
|  | 0,0002604 | 3,031E-06 | 1,005E-05 |
|  | 0,0003958 | 8,961E-07 | 1,314E-05 |

| *XACT* expression | WT | KO | INV |
| --- | --- | --- | --- |
|  | 0,0013617 | 0,0010517 | 0,0004434 |
|  | 0,0011319 | 0,0014975 | 0,0014031 |
|  | 0,0010019 | 0,0010768 | 0,0013023 |

- Figure 4C

| XACT | WT | | KO T113.3 | | | INV T113.3 | | | |
| --- | --- | --- | --- | --- | --- | --- | --- | --- | --- |
| Day 0 | 1 | 1 | 1,321973 | 0,4265019 | 0,629344 | 1,167408 | 0,5181553 | 1,988631 | 0,8868226 |
| 1 | 1,714507 | 2,052948 | 1,600212 |  | 1,015388 | 1,754036 | 1,179518 | 2,181659 | 1,293754 |
| 2 | 2,188436 | 2,84055 | 3,319181 |  | 1,208651 | 2,240407 | 1,585288 | 3,401211 | 1,484343 |
| 3 | 1,329457 | 2,449893 | 1,510222 | 0,32037 | 0,9153192 | 1,36385 | 0,8244382 | 1,539754 | 0,7338545 |
| 4 | 0,6123745 | 0,5606493 | 0,4247457 | 0,0768742 | 0,3300252 | 0,1204311 | 0,2039201 | 0,2973926 | 0,2596853 |
| 5 | 0,1582198 | 0,22439 | 0,1420187 | 0,0118914 | 0,061443 | 0,1862928 | 0,0811261 | 0,1999646 | 0,0484742 |
| 6 | 0,0406525 | 0,1568689 | 0,1149553 | 0,0233309 | 0,0841308 | 0,1387242 | 0,1050429 | 0,2383905 | 0,0540268 |
| 7 | 0,0497416 | 0,1142196 | 0,1023103 | 0,0191036 | 0,0192477 | 0,1205571 | 0,030923 | 0,1368867 | 0,0230895 |
| 8 | 0,0380539 | 0,1179599 | 0,1046813 | 0,0181293 | 0,0219483 | 0,100598 | 0,0238415 | 0,1000082 | 0,0276233 |
| 9 | 0,0462948 | 0,125662 | 0,1197989 | 0,0322485 | 0,0144783 | 0,1422733 | 0,0417309 | 0,1527633 | 0,0346006 |
| 10 | 0,0302239 | 0,151254 | 0,1338421 | 0,0273979 | 0,0157853 | 0,171764 | 0,0336545 | 0,116843 | 0,0297786 |

**Supp figure 4**

- Supp figure 4A

| OCT4 | no_LNA | LNA_SCR | LNA_X1 | LNA_X2 | LNA_T1 | LNA_T2 |
| --- | --- | --- | --- | --- | --- | --- |
| n=1 | 1 | 1,698191 | 1,049717 | 0,6142935 | 1,149494 | 1,014662 |
| n=2 | 1 | 0,9676125 | 0,9356237 | 0,9800999 | 1,650039 | 0,5817621 |
| n=3 | 1 | 0,8993783 | 0,857376 | 0,9362723 | 0,9944702 |  |
| n=4 | 1 | 1,184272 | 0,8010699 | 0,9050065 | 1,19914 | 1,039579 |
| n=5 | 1 | 0,7852733 | 0,8682309 | 1,010111 | 0,61385 | 1,230921 |
| n=6 | 1 | 0,8025684 | 0,8637575 | 1,481032 | 1,154823 | 0,8523263 |

| NODAL | no_LNA | LNA_SCR | LNA_X1 | LNA_X2 | LNA_T1 | LNA_T2 |
| --- | --- | --- | --- | --- | --- | --- |
| n=1 | 1 | 0,8669379 | 1,150691 | 0,5532486 | 0,9968854 | 0,9576033 |
| n=2 | 1 | 0,8435248 | 0,7285001 | 0,7164809 | 1,016423 | 0,6147196 |
| n=3 | 1 | 0,9753554 | 0,7422618 | 0,8729676 | 0,9150991 |  |
| n=4 | 1 | 1,057018 | 0,8675387 | 0,9214642 | 0,9050065 | 1,53581 |
| n=5 | 1 | 0,6304165 | 0,6525071 | 0,8865764 | 0,6000541 | 1,330862 |
| n=6 | 1 | 0,8530506 | 1,006719 | 1,447762 | 1,058628 | 1,311317 |

- Supp figure 4C

| OCT4 | WT | KO | INV |
| --- | --- | --- | --- |
|  | 0,2182816 | 0,2179531 | 0,2176272 |
|  | 0,2429277 | 0,2410699 | 0,1908753 |
|  | 0,1950343 | 0,2186557 | 0,1866653 |

| NODAL | WT | KO | INV |
| --- | --- | --- | --- |
|  | 0,0010555 | 0,0007258 | 0,0005848 |
|  | 0,0005952 | 0,001547 | 0,000401 |
|  | 0,0003176 | 0,0007438 | 0,0003789 |

| AMOT | WT | KO | INV |
| --- | --- | --- | --- |
|  | 0,001185 | 0,000963 | 0,001025 |
|  | 0,001232 | 0,001254 | 0,001272 |

| HTR2C | WT | KO | INV |
| --- | --- | --- | --- |
|  | 0,002436 | 0,00169 | 0,002149 |
|  | 0,000932 | 0,002809 | 0,000833 |

| ATRX | WT | KO | INV |
| --- | --- | --- | --- |
|  | 0,012542 | 0,010476 | 0,012494 |
|  | 0,016151 | 0,014111 | 0,010238 |

- Supp Figure 4D

| T113.3 | WT | | KO | | | Inv | | | |
| --- | --- | --- | --- | --- | --- | --- | --- | --- | --- |
| Day 0 | 1 | 1 | 0,0025979 | 0,0019437 | 0,0008134 | 0,0051487 | 0,0020353 | 0,0110928 | 0,0045428 |
| 1 | 0,7666298 | 0,9489012 | 0,0027021 | 0,0006524 | 0,0014324 | 0,0207623 | 0,0066293 | 0,0116541 | 0,009228 |
| 2 | 0,7096514 | 0,930077 | 0,0043548 | 0,0070536 | 0,0055713 | 0,0127334 | 0,0018733 | 0,0091228 | 0,0043733 |
| 3 | 0,3913812 | 0,567019 | 0,0046002 | 0,0006306 | 0,002631 | 0,007794 | 0,0062534 | 0,0078096 | 0,0042325 |
| 4 | 0,1974714 | 0,1621645 | 0,0049724 | 0,0008319 | 0,004483 | 0,0255402 | 0,0044673 | 0,004732 | 0,0046956 |
| 5 | 0,0766398 | 0,0582696 | 0,0047054 | 0,0006527 | 0,0026721 | 0,009848 | 0,0052824 | 0,0044114 | 0,0049238 |
| 6 | 0,0053626 | 0,0605367 | 0,0032965 | 0,0005813 | 0,0006208 | 0,00663 | 0 | 0,0024277 | 0,0010286 |
| 7 | 0,0192599 | 0,021041 | 0,0041476 | 0,0008447 | 0,0016089 | 0,0070762 | 0,0015889 | 0,0042435 | 0,0051706 |
| 8 | 0,0099574 | 0,0168742 | 0,0047141 | 0,0004498 | 0,0021998 | 0,0031275 | 0,0020385 | 0,0040237 | 0,0013086 |
| 9 | 0,0055565 | 0,0163035 | 0,002813 | 0,0008562 | 0,0019998 | 0,0041938 | 0,0017541 | 0,0018236 | 0,0019176 |
| 10 | 0,0042256 | 0,0052412 | 0,0055475 | 0,0005072 | 0,0027502 | 0,0058533 | 0,0009751 | 0,0038594 | 0,000928 |

| HAND1 | WT | | KO | | | INV | | |
| --- | --- | --- | --- | --- | --- | --- | --- | --- |
| Day 0 | 1 | 1 | 0,8370321 | 0,8931552 | 1,665393 | 1,539841 | 1,397551 | 0,8721318 |
| 1 | 1,118693 | 1,880979 | 0,9405156 | 1,24725 | 0,4491378 | 1,506987 | 2,305302 | 0,9142452 |
| 2 | 2,646293 | 17,32038 | 2,64709 | 3,369292 | 1,581727 | 15,54006 | 11,37479 | 11,40767 |
| 3 | 85,34087 | 148,4963 | 83,96255 | 89,22699 | 178,6121 | 142,4683 | 151,6813 | 98,54919 |
| 4 | 99,54373 | 241,2732 | 121,3221 | 136,6301 | 355,6306 | 209,0962 | 275,3596 | 137,6478 |
| 5 | 102,9457 | 198,2153 | 89,62684 | 103,4572 | 69,00583 | 174,5569 | 210,2855 | 110,1553 |
| 6 | 62,31089 | 101,5987 | 52,81416 | 43,80698 |  | 97,02294 | 144,2352 | 31,93496 |
| 7 | 26,64055 | 60,7532 | 14,10891 | 19,50768 | 52,20713 | 44,09694 | 39,98139 | 22,65367 |
| 8 | 13,93265 | 32,01913 | 8,997744 | 12,5923 | 53,21598 | 32,14616 | 23,56732 | 14,02224 |
| 9 | 7,822504 | 25,29994 | 6,112947 | 12,06885 | 121,7946 | 21,52151 | 18,89486 | 8,602156 |
| 10 | 5,887259 | 15,08843 | 3,486872 | 4,086259 | 20,16992 | 10,4573 | 13,52983 | 3,621019 |

| OCT4 | WT | | KO | | | Inv | | | |
| --- | --- | --- | --- | --- | --- | --- | --- | --- | --- |
| Day 0 | 1 | 1 | 0,9723882 | 0,5521964 | 0,977203 | 1,271412 | 1,061404 | 1,149212 | 1,042672 |
| 1 | 1,049793 | 0,8976144 | 1,149594 | 0,5969098 | 0,9682856 | 1,08478 | 0,965148 | 0,8606148 | 0,9730453 |
| 2 | 1,239987 | 1,243154 | 1,179027 | 0,963325 | 1,256553 | 1,433986 | 0,9458076 | 1,433109 | 0,9612538 |
| 3 | 1,223398 | 1,136044 | 0,8596653 | 0,4746934 | 1,36448 | 1,328517 | 1,336957 | 1,048972 | 1,219414 |
| 4 | 0,6408569 | 0,5252095 | 0,5058444 | 0,2084035 | 0,8115551 |  | 0,8109588 | 0,4830853 | 0,7632742 |
| 5 | 0,1679571 | 0,1525816 | 0,132316 | 0,0711436 | 0,1730658 | 0,1453364 | 0,1837413 | 0,1218104 | 0,1670708 |
| 6 | 0,0102983 | 0,046954 | 0,071245 | 0,0164201 | 0,0165793 | 0,0783883 | 0,0136334 | 0,0974876 | 0,0193766 |
| 7 | 0,0210379 | 0,0130388 | 0,0123048 | 0,0050314 | 0,0127402 | 0,0150358 | 0,0135182 | 0,0138125 | 0,011258 |
| 8 | 0,0165703 | 0,0093836 | 0,0093742 | 0,0052678 | 0,0097026 | 0,010393 | 0,0104051 | 0,009636 | 0,0093568 |
| 9 | 0,0143964 | 0,0111734 | 0,0110291 | 0,0057711 | 0,0098168 | 0,0129113 | 0,0121155 | 0,010624 | 0,0115803 |
| 10 | 0,0124032 | 0,0112924 | 0,0105091 | 0,0047721 | 0,0078049 | 0,0119441 | 0,0107598 | 0,009666 | 0,0077152 |

| GATA6 | WT | | KO | | | Inv | | | |
| --- | --- | --- | --- | --- | --- | --- | --- | --- | --- |
| Day 0 | 1 | 1 | 0,8415548 | 1,082779 | 1,311069 | 1,153545 | 0,6999857 | 1,498898 | 2,162356 |
| 1 | 55,27865 | 42,99718 | 25,64655 | 7,400863 | 65,17182 | 50,93097 | 65,01365 | 41,27415 | 72,90117 |
| 2 | 92,72543 | 20,15288 | 25,82296 | 2,736007 | 94,86652 | 18,87568 | 80,2134 | 39,61942 | 99,42352 |
| 3 | 89,59628 | 97,98756 | 72,01165 | 34,78326 | 101,0905 | 65,59192 | 98,58764 | 70,46346 | 114,8095 |
| 4 | 162,7952 | 112,1428 | 114,7202 | 97,06644 | 163,5673 | 8,919303 | 164,9096 | 120,3908 | 173,7814 |
| 5 | 246,3453 | 162,4808 | 174,2074 | 70,6363 | 203,3579 | 221,2398 | 262,2155 | 233,153 | 275,8322 |
| 6 | 405,689 | 184,3087 | 134,6983 | 149,0104 | 698,0966 | 150,6575 | 752,9557 | 87,5358 | 359,6153 |
| 7 | 438,6302 | 322,0721 | 335,7485 | 254,2639 | 323,7511 | 379,0504 | 338,0271 | 528,1178 | 422,0839 |
| 8 | 408,9718 | 412,0572 | 355,8733 | 294,0704 | 408,0116 | 379,3964 | 407,0418 | 517,6602 | 407,715 |
| 9 | 347,3841 | 358,2529 | 335,5244 | 299,8759 | 178,8186 | 437,6842 | 504,1678 | 478,6971 | 542,1521 |
| 10 | 170,3895 | 364,3719 | 307,7505 | 216,53 | 269,1975 | 358,0337 | 307,1546 | 325,3111 | 380,1837 |

**Figure 5**

- Figure 5B

ΔCTCF

| *XACT* expression | WT | KO | INV |
| --- | --- | --- | --- |
|  | 0,007643 | 0,005865 | 0,005235 |
|  | 0,007633 | 0,01221 | 0,012436 |
|  | 0,005722 | 0,015522 | 0,004353 |
|  | 0,005687 | 0,015629 | 0,003749 |
|  |  | 0,008799 |  |
|  |  | 0,008507 |  |

| *T113*.3 expression | WT | KO | INV |
| --- | --- | --- | --- |
|  | 0,010694 | 0,009485 | 0,011756 |
|  | 0,010849 | 0,011639 | 0,011818 |
|  | 0,017338 | 0,015326 | 0,009962 |
|  | 0,009754 | 0,011895 |  |
|  |  | 0,010534 |  |
|  |  | 0,012712 |  |

ΔTFB

| *XACT* expression | WT | KO | INV |
| --- | --- | --- | --- |
|  | 0,013065 | 0,001858 | 0,009979 |
|  | 0,009445 | 0,001249 | 0,008897 |
|  | 0,005927 | 0,001271 |  |
|  | 0,007983 | 0,001788 |  |
|  | 0,008555 | 0,001634 |  |
|  | 0,005725 | 0,002403 |  |

| *T113*.3 expression | WT | KO | INV |
| --- | --- | --- | --- |
|  | 0,008972 | 0,0000652 | 0,009987 |
|  | 0,012835 | 0,0000403 | 0,008616 |
|  | 0,016806 | 0,0000422 |  |
|  | 0,011413 | 0,000053 |  |
|  | 0,011374 | 0,0000595 |  |
|  | 0,00973 | 0,0000482 |  |

- Figure 5C

| *XACT* expression | no_si | siSCR | siSOX2 | siNANOG | siOCT4 | siSOX2/OCT4 |
| --- | --- | --- | --- | --- | --- | --- |
|  | 1 | 1,318041 | 0,6972711 | 1,357972 | 1,229917 | 0,396986 |
|  | 1 | 0,5207987 | 0,1231937 | 0,4113556 | 0,9883534 | 0,1581338 |
|  | 1 | 0,7842085 | 0,7484589 | 1,14337 | 1,442976 | 0,3823967 |
|  | 1 | 1,24083 | 0,5710204 | 1,396596 | 0,9633619 | 0,3127488 |
|  | 1 | 0,8622525 | 0,5197092 | 1,031829 | 0,942339 | 0,1197159 |

| *T113*.3 expression | no_si | siSCR | siSOX2 | siNANOG | siOCT4 | siSOX2/OCT4 |
| --- | --- | --- | --- | --- | --- | --- |
|  | 1 | 1,132701 | 0,8992761 | 1,177991 | 1,62302 | 0,1867206 |
|  | 1 | 1,050909 | 1,041818 | 1,00627 | 2,605016 | 0,24108 |
|  | 1 | 0,8512216 | 1,330387 | 1,214009 | 2,709596 | 0,3530405 |
|  | 1 | 0,6623801 | 0,5695908 | 0,7914923 | 1,279985 | 0,1059574 |
|  | 1 | 1,028351 | 0,8252408 | 0,8733398 | 1,321613 | 0,05894246 |

- Figure 5C


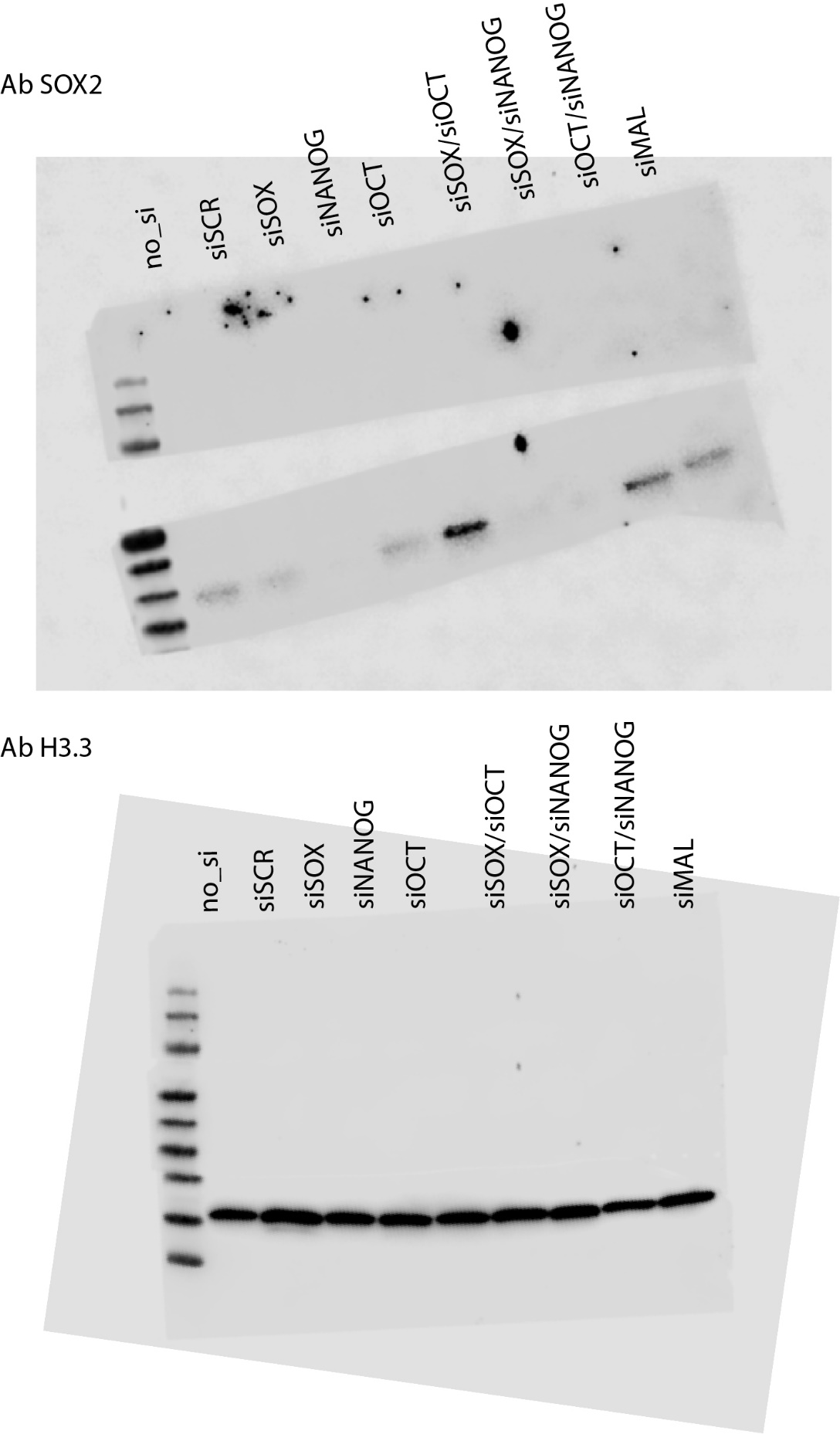


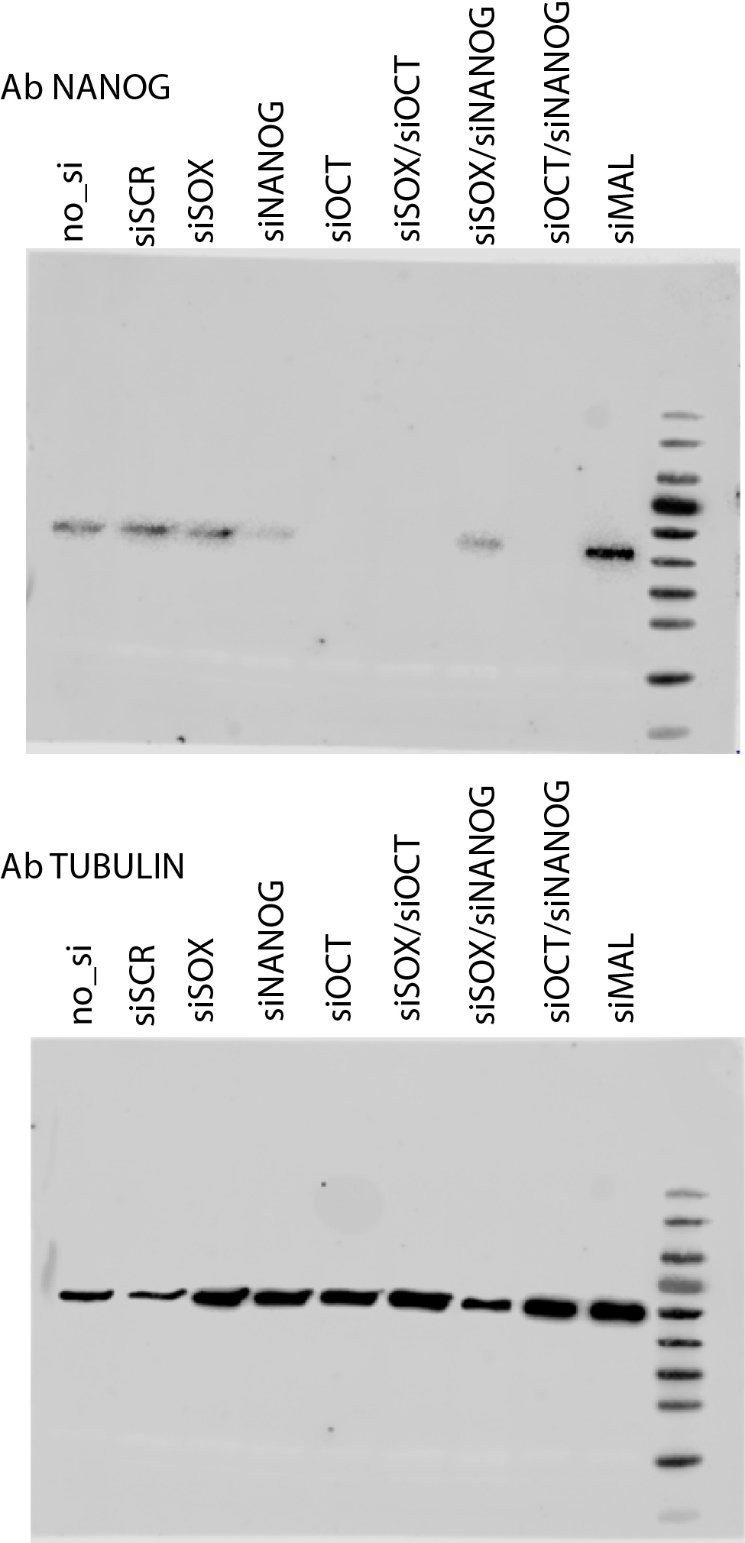


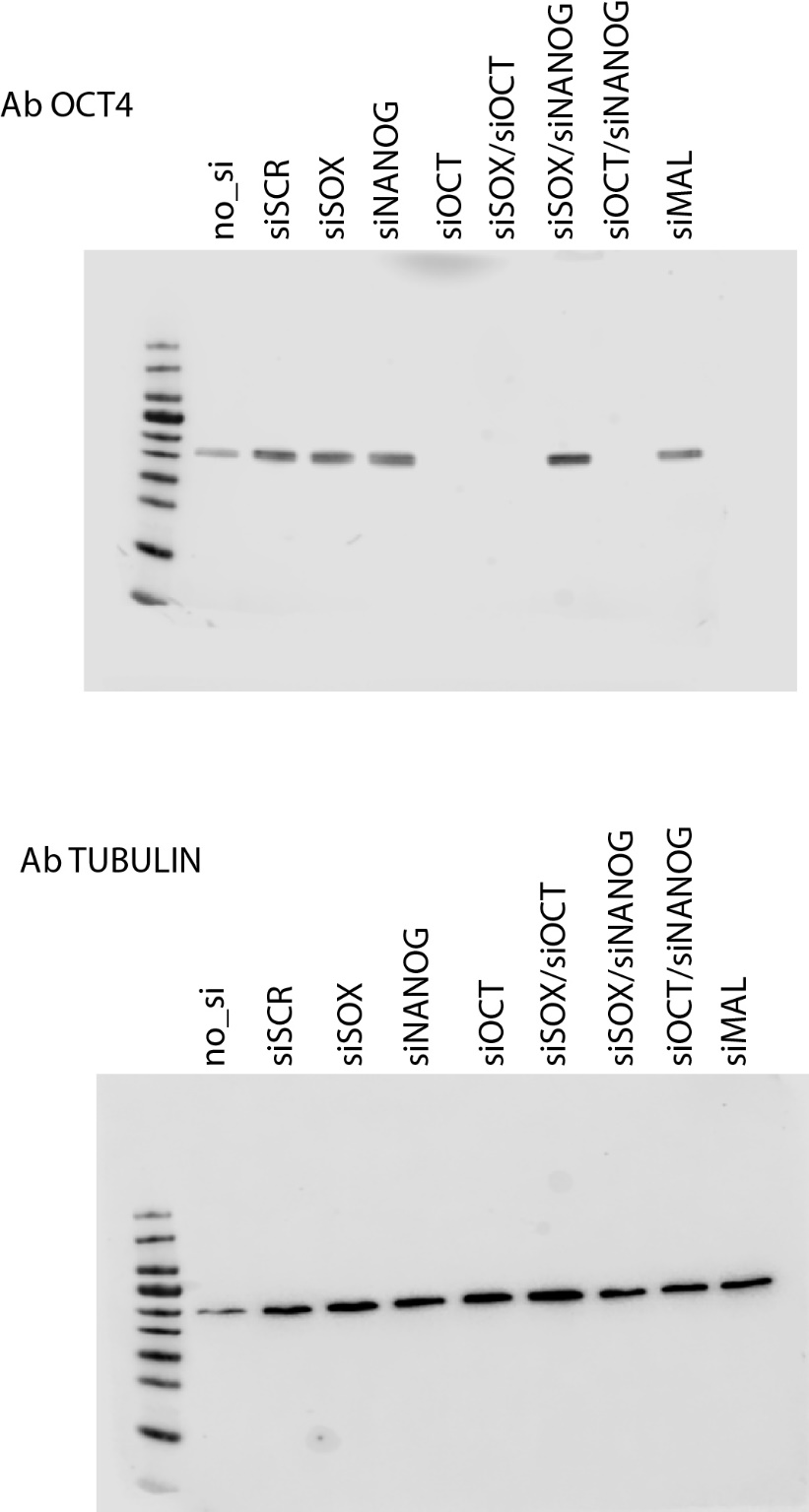


**Supp Figure 5**

- Supp figure 5C

ΔCTCF

| OCT4 expression | WT | DEL | INV |
| --- | --- | --- | --- |
|  | 0,528717 | 0,586023 | 0,523454 |
|  | 0,449857 | 0,544936 | 0,5988 |
|  | 0,550526 | 0,791122 |  |
|  | 0,638612 | 0,638363 |  |
|  | 0,50881 | 0,594654 |  |
|  | 0,535158 | 0,549586 |  |

| NANOG expression | WT | DEL | INV |
| --- | --- | --- | --- |
|  | 0,088285 | 0,080452 | 0,059605 |
|  | 0,062134 | 0,090406 | 0,097683 |
|  | 0,067092 | 0,085985 | 0,066487 |
|  | 0,076353 | 0,080425 | 0,062154 |
|  |  | 0,089847 |  |
|  |  | 0,082332 |  |

ΔTFB

| OCT4 expression | WT | DEL | INV |
| --- | --- | --- | --- |
|  | 0,652106 | 0,665298 | 0,56986 |
|  | 0,618533 | 0,600782 | 0,600912 |
|  | 0,515328 | 0,501921 |  |
|  | 0,63233 | 0,596965 |  |
|  | 0,558601 | 0,612155 |  |
|  | 0,560635 | 0,620243 |  |

| NANOG expression | WT | DEL | INV |
| --- | --- | --- | --- |
|  | 0,098696 | 0,071887 | 0,078761 |
|  | 0,069115 | 0,05909 | 0,068432 |
|  | 0,063302 | 0,058032 |  |
|  | 0,062523 | 0,086236 |  |
|  | 0,093464 | 0,092819 |  |
|  | 0,076777 | 0,083627 |  |

- Supp figure 5D

*XACT* expression

| no_si | siSCR | siSOX2 | siNANOG | siOCT4 | siSOX2/OCT4 | siSOX2/NANOG | siOCT4/NANOG |
| --- | --- | --- | --- | --- | --- | --- | --- |
| 1 | 1,318041 | 0,6972711 | 1,357972 | 1,229917 | 0,396986 | 0,9874001 | 0,5386678 |
| 1 | 0,5207987 | 0,1231937 | 0,4113556 | 0,9883534 | 0,1581338 | 0,1754565 | 0,5204456 |
| 1 | 0,7842085 | 0,7484589 | 1,14337 | 1,442976 | 0,3823967 | 0,9284479 | 0,8960884 |
| 1 | 1,24083 | 0,5710204 | 1,396596 | 0,9633619 | 0,3127488 | 1,102807 | 1,321751 |
| 1 | 0,8622525 | 0,5197092 | 1,031829 | 0,942339 | 0,1197159 | 0,6542892 | 0,3881324 |

*T113.3* expression

| no_si | siSCR | siSOX2 | siNANOG | siOCT4 | siSOX2/OCT4 | siSOX2/NANOG | siOCT4/NANOG |
| --- | --- | --- | --- | --- | --- | --- | --- |
| 1 | 1,132701 | 0,8992761 | 1,177991 | 1,62302 | 0,1867206 | 0,8876378 | 0,5520628 |
| 1 | 1,050909 | 1,041818 | 1,00627 | 2,605016 | 0,24108 | 1,152807 | 1,678235 |
| 1 | 0,8512216 | 1,330387 | 1,214009 | 2,709596 | 0,3530405 | 1,39952 | 1,668937 |
| 1 | 0,6623801 | 0,5695908 | 0,7914923 | 1,279985 | 0,1059574 | 0,9014366 | 0,8491288 |
| 1 | 1,028351 | 0,8252408 | 0,8733398 | 1,321613 | 0,05894246 | 0,8058288 | 0,9843931 |

*GATA6* expression

| no_si | siSCR | siSOX2 | siNANOG | siOCT4 | siSOX2/OCT4 | siSOX2/NANOG | siOCT4/NANOG |
| --- | --- | --- | --- | --- | --- | --- | --- |
| 1 | 0,6283812 | 2,53231 | 11,45379 | 0,9457038 | 17,3871 | 0,9290016 | 3,487771 |
| 1 | 0,3096082 | 0,6352927 | 9,002968 | 0,4838849 | 8,679631 | 0,5925115 | 3,136121 |
| 1 | 0,2256018 | 0,9960746 | 9,418183 | 0,6933973 | 1,149062 | 0,7067105 | 3,182613 |
| 1 | 0,6617367 | 0,1219469 | 17,88047 | 0,9497245 | 74,20908 | 2,114377 | 13,55497 |
| 1 | 1,595372 | 8,107937 | 146,959 | 6,162062 | 250,9069 | 7,263709 | 68,52074 |

*PAX6* expression

| no_si | siSCR | siSOX2 | siNANOG | siOCT4 | siSOX2/OCT4 | siSOX2/NANOG | siOCT4/NANOG |
| --- | --- | --- | --- | --- | --- | --- | --- |
| 1 | 1,39173 | 1,215794 | 346,5964 | 4,073804 | 111,4451 | 2,090025 | 470,2808 |
| 1 | 1,528467 | 0,7209663 | 535,9038 | 7,725247 | 187,563 | 4,571637 | 582,1724 |
| 1 | 2,615075 | 1,01426 | 882,1905 | 1,684307 | 808,8995 | 1,699385 | 804,5012 |
| 1 | 1,756343 | 1,816028 | 120,6631 | 3,872876 | 25,61304 | 3,422316 | 236,1132 |
| 1 | 5,167467 | 4,921392 | 318,4818 | 8,307689 | 38,51955 | 10,71913 | 402,5193 |

**Datasets used in this study**

| **RNA-seq** | | | |
| --- | --- | --- | --- |
| H1 hESCs | ENCODE | Fig. 1D / Sup. Fig. 1 | GSM758573 |
| Rhesus iPSCs | IHEC | Sup. Fig. 1 | IHECRE00000812.1 |
| Chimpanzee iPSCs | IHEC | Sup. Fig. 1 | IHECRE00000673.1 |
| Human iPSCs | IHEC | Sup. Fig. 1 | IHECRE00000914.1 |

| **scRNA-seq** | |
| --- | --- |
| Yan et al., 2013 | GSE36552 |
| Xue et al., 2013 | GSE44183 |
| Blakeley et al., 2015 | GSE66507 |
| Petropoulous et al., 2016 | E-MTAB-3929 |

| **ATAC-seq** | | | |
| --- | --- | --- | --- |
| H1 hESCs | CISTROME | Fig. 5A | GSM2386576 |

| **ChIP-Seq** | | | | |
| --- | --- | --- | --- | --- |
| H3K4me3_H1 hESCs | ENCODE |  | Fig. 1D | GSM733657 |
| H3K4me3_H1 hESCs | CISTROME | Lister et al., 2009 | Fig. 5A | GSM409308 |
| H3K27ac_H1 hESCs | CISTROME | Lister et al., 2009 | Fig. 5A | GSM466732 |
| CTCF_H1 | CISTROME | Dixon et al., 2015 | Fig. 5A | GSM1267206 |
| OCT4_H1 | CISTROME | Gertz et al., 2013 | Fig. 5A | GSM803438 |
| SOX2_H1 | CISTROME | Lister et al., 2009 | Fig. 5A | GSM456570 |
| NANOG_H1 | CISTROME | Lister et al., 2009 | Fig. 5A | GSM456571 |
| H3K4me3_IMR90 | CISTROME | Lister et al., 2009 | Sup. Fig. 5A | GSM521901 |
| H3K27ac_IMR90 | CISTROME | Lister et al., 2009 | Sup. Fig. 5A | GSM521887 |
| NANOG_H1 |  | Lyu et al., 2018 | Sup. Fig. 6C | GSM2816625 |
| OCT4_H1 |  | Lyu et al., 2018 | Sup. Fig. 6C | GSM2816629 |
| IgG_H1 |  | Lyu et al., 2018 | Sup. Fig. 6C | GSM2816613 |
